# Protocol for chronic electroencephalography, fiber photometry, and silicon probe recordings in the Syrian hamster

**DOI:** 10.1016/j.xpro.2026.104863

**Published:** 2026-10-05

**Authors:** Fuki Yamashita, Ren Y. Sato, Yasutaka Mukai, Hiroaki Nishimura, Sarah Dagher, Shimpei Ishiyama, Miyu Kimata, Riho Kobayashi, Hiroaki Norimoto

**Affiliations:** 1 Graduate School of Science, Nagoya University, Nagoya, Aichi 464-8602, Japan; 2 Max-Planck-Institut für Stoffwechselforschung, 50931 Köln, Germany; 3 Department of Neuropeptide Research in Psychiatry, Research Group for Neurobiology of Positive Emotions, Central Institute of Mental Health, 68159 Mannheim, Germany

**Keywords:** Neuroscience, Cognitive Neuroscience, Behavior

## Abstract

The Syrian hamster (*Mesocricetus auratus*) exhibits a unique metabolic physiology, including the ability to enter hypometabolic states. While this species represents a powerful model for studying the neuronal basis of metabolic control and brain-state transitions, its use is limited due to the lack of detailed protocols for chronic neuronal measurements. Here, we present a step-by-step protocol for chronic surgical implantation and multimodal neuronal recordings in Syrian hamsters. We describe steps for electroencephalography (EEG), fiber photometry, and multi-channel silicon probe recordings.

## Highlights

• Steps for multimodal neuronal recordings in freely behaving Syrian hamsters
• Techniques for stable skull exposure and implantation adapted for Syrian hamsters
• Procedures for reusable silicon probe recovery and troubleshooting chronic recordings

## Abstract (editor highlights)

Publisher’s note: Undertaking any experimental protocol requires adherence to local institutional guidelines for laboratory safety and ethics.

## Graphical abstract

**Figure:**
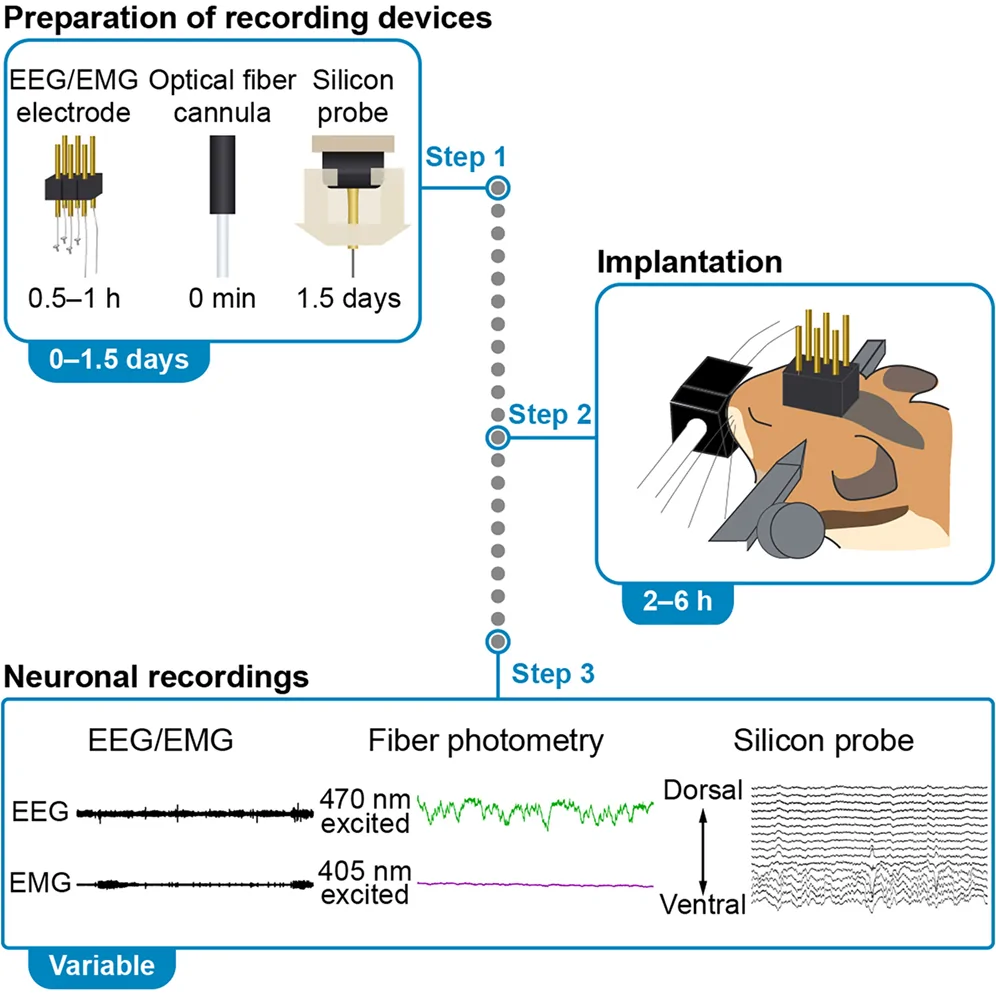

## Before you begin

The Syrian hamster (*Mesocricetus auratus*) is widely used as an experimental model in sleep, circadian rhythm, and metabolic research.^1^ This species exhibits robust nocturnal behavioral rhythms and can enter hypometabolic states characterized by marked reductions in body temperature and metabolic activity under specific environmental conditions.^2^^,^^3^ These unique physiological properties make the Syrian hamster a powerful model for investigating the neuronal basis of metabolic regulation and brain state transitions.

In addition to its physiological advantages, the Syrian hamster is also well suited for chronic neuronal recordings. Compared with mice, its larger brain size enables precise stereotaxic manipulation of target brain regions and stable implantation of various chronic recording devices. In contrast to rats, its smaller body size allows efficient experimental design even under limited housing conditions.

### Innovation

Despite the physiological and practical advantages of Syrian hamsters, detailed surgical procedures and analytical protocols for chronic neuronal recordings in this species remain insufficiently established, limiting its broader use in systems neuroscience research. Although the general principles of chronic electrophysiological recordings are shared across rodents, direct adoption of mouse or rat protocols is often challenging because Syrian hamsters differ in cranial anatomy, stereotaxic coordinates, and several perioperative considerations. As a result, researchers often need to adapt existing protocols through trial and error, which can reduce reproducibility and hinder the adoption of this experimental model.

In this protocol, we present step-by-step procedures for chronic surgical implantation and multimodal neuronal recordings in Syrian hamsters, including electroencephalography (EEG), fiber photometry, and multi-channel silicon probe recordings. The protocol combines practical guidance for hamster-specific surgical procedures with established recording methodologies, providing a comprehensive resource for investigators seeking to perform chronic neuronal recordings in this species.

### Institutional permissions (if applicable)

All animal experiments described in this protocol were approved by the Animal Care and Use Committee of Nagoya University (S260007-003) and were conducted in accordance with institutional guidelines.

## Key resources table

**Table:** 

REAGENT or RESOURCE	SOURCE	IDENTIFIER
**Antibodies**		

Chicken anti-GFP antibody (1:2000)	Abcam Limited	Cat#ab13970; RRID: AB_300798
Goat anti-Chicken IgY antibody, Alexa 488 conjugated (1:500)	Thermo Fisher Scientific K.K.	Cat#A-11039; RRID: AB_2534096

**Bacterial and virus strains**		

AAV2/9-hSyn-NE2m	WZ Biosciences, Inc.	YL003008-AV9

**Chemicals, peptides, and recombinant proteins**		

Isoflurane	Viatris Pharmaceuticals Japan G.K.	Isoflurane inhalation solution “VTRS”
Saline	N/A	N/A
Meloxicam	Kyoritsu Seiyaku Corporation	MELOXICAM 2% injection
Ophthalmic ointment	Pfizer Japan Inc.	Neo-Medrol EE Ointment
Ethanol	Yamazen pharmaceutical	Shoeta Value
Iodine	Meiji Seika Pharma Co., Ltd.	Povidone-Iodine Scrub Solution 7.5% “MEIJI”
0.1% Adrenaline	DAIICHI SANKYO COMPANY, LIMITED	BOSMIN SOLUTION
Lidocaine	Nissin Pharmaceutical Co., Ltd.	Lidocaine Pump Spray 8% “NISSIN”
Terramycin	JNTL Consumer Health K.K.	Terramycin Ointment a
Dexamethasone	FUJIFILM Wako Pure Chemical Corporation	047–18863
Dimethyl Sulfoxide	FUJIFILM Wako Pure Chemical Corporation	049–07213
DiI	FUJIFILM Wako Pure Chemical Corporation	045–33421
Glyceol	TAIYO Pharma Co., Ltd.	GLYCEOL Injection 200 mL
Dura-Gel	Cambridge NeuroTech	Dura-Gel
Terg-a-zyme® enzyme detergent	Sigma-Aldrich Co. LLC	Z273287
Ultrapure water	N/A	N/A
Paraformaldehyde	FUJIFILM Wako Pure Chemical Corporation	162–16065

**Deposited data**		

STL files for the microdrive design and source data underlying the figures	This paper	Mendeley Data: https://data.mendeley.com/datasets/n64gw7ywy4/1.

**Experimental models: Organisms/strains**		

Syrian hamster (wild-type, ≥7 weeks old, both sexes)	Japan SLC, Inc.	Slc:Syrian

**Software and algorithms**		

Bonsai 2.9.0	Bonsai Foundation CIC	https://bonsai-rx.org/docs/articles/installation.html
LabVIEW 2023 Q3	National Instruments Corp.	https://www.ni.com/en/support/downloads/software-products/download.labview.html#487445
Open Ephys GUI 0.6.4	Open Ephys	https://open-ephys.org/gui
PMT2100 Control version 4.0	Thorlabs Inc.	https://www.thorlabs.com/software-pages/pmt
MATLAB R2023b	MathWorks	https://www.mathworks.com/products/matlab.html

**Other**		

1 mL syringe	Terumo Corporation	SS-10M2913A
Kimwipes	NIPPON PAPER CRECIA CO., LTD.	Kimwipes S-200
Anesthesia machine	KIMURAMED Company Limited	Anesthesia Machine for veterinary, TASUKE
Heating pad	Bio Research Center Co., Ltd	BWT-100A
Stereotaxic apparatus	NARISHIGE SCIENTIFIC INSTRUMENT LAB.	SR-6R-HT
Ear bars	NARISHIGE SCIENTIFIC INSTRUMENT LAB.	EB-3A
Incisor bar	NARISHIGE SCIENTIFIC INSTRUMENT LAB.	RA-6N
Nose holder	NARISHIGE SCIENTIFIC INSTRUMENT LAB.	GM-3
Stereotaxic manipulator	NARISHIGE SCIENTIFIC INSTRUMENT LAB.	SMM-100
Implant holder	NARISHIGE SCIENTIFIC INSTRUMENT LAB.	SMM-HE
Scissors	Fine Science Tools Inc	14058–11
Forceps	Nazme Co. Ltd.	NAPOX A-14
Forceps	Nazme Co. Ltd.	NAPOX A-5
Forceps	Fine Science Tools Inc	11252–20
Tissue clamp	Fine Science Tools Inc	18051–28
Scalpel	FEATHER Safety Razor Co., Ltd.	Feather Scalpel Sterilized No.14
Cotton swab	Suncloud	261–4272
Soldering iron	HAKKO Corporation	FX-600
Solder	TAIYO ELECTRIC IND.CO.,LTD.	SD-89
Flux	TAIYO ELECTRIC IND.CO.,LTD.	BS-45
Stainless screw	SUNCO INDUSTRIES Co.,Ltd.	CSPPN1-SUS-M1-2
Stainless wire	A-M Systems	791400
Pin header	Harwin	M20-9980545
Miniature wire cutter	HOZAN TOOL INDUSTRIAL CO., LTD.	N-31
Screwdriver	ENGINEER INC.	DM-22
Drill bit	Fine Science Tools Inc	19007–07
Drill	ARGOFILE JAPAN LIMITED	SBH35-STW
Heat-shrink tubing	Sumitomo Electric Industries, Ltd.	SUMITUBE C 3×0.2 Black
Suture with needle	Alfresa Pharma Corporation	HT2004SB45-AF8
Super-Bond Catalyst V	Sun Medical Company, Ltd.	204610580
Super-Bond Quick Monomer	Sun Medical Company, Ltd.	204610399
Super Bond Polymer (Bulk-mix teeth color)	Sun Medical Company, Ltd.	204610564
Super-Bond C&B Red Activator	Sun Medical Company, Ltd.	204610460
Super-Bond C&B Green Activator	Sun Medical Company, Ltd.	204610461
Transbond XT Light Cure Adhesive	3M Unitek	712–036
Wireless dental curing light	Amasie	N/A
Aron Alpha A “Sankyo”	TOAGOSEI CO.,LTD.	081–019311
Dental acrylic resin	SHOFU INC.	Provinice
Shielded cable	Oyaide Elec. Co., Ltd.	3109
Commutator	Moog Inc.	AC6023-12
Adapter board	Intan Technologies	#C3418
Preamplifier	Intan Technologies	#C3334
Open Ephys Acquisition Board	Open Ephys	Open Ephys Acquisition Board
SPI cable (0.9 m)	Intan Technologies	#C3203
SPI cable (1.8 m)	Intan Technologies	#C3206
Camera	Teledyne Vision Solutions	BFS-U3-16S2M-CS
Lens	Theia Technologies	SL183M
USB cable	Teledyne Vision Solutions	ACC-01–2300
I/O Board	Open Ephys	OEPS-6501
BNC cable (3m)	CONTEC CO., LTD.	BNC-B300
BNC isolated receptacle	TO-CONNE CO., LTD.	BNC-31-10L
GPIO cable	Teledyne Vision Solutions	ACC-01–3010
Connector	Hirose Electric Co., Ltd.	HR10A-7R-6S(73)
Connector receptacle	Hirose Electric Co., Ltd.	HR10A-7R-6P(73)
Perfboard	AKIZUKI DENSHI TSUSHO CO., LTD.	AE-C5 1.2t
3-pole green vertical terminal block	AKIZUKI DENSHI TSUSHO CO., LTD.	TB112-2-3-E−1
CPU	Intel Corporation	13th Gen Intel(R) Core(TM) i7-13700
SSD	Micron Technology, Inc.	crucial BX500 CT1000BX500SSD1JP
HDD	Western Digital Corporation	WD40EZZX
Wire	PLUS Corporation	TF-800
Masking tape	Teraoka Seisakusho co.,Ltd.	4140
Micropipette Puller	RWD Life Science Co.,LTD	MP-500
Standard Glass Capillaries	World Precision Instruments	1B100F-4
1 mL syringe without needle	Terumo Corporation	SS-01T
Luer fitting	Nordson MEDICAL	VRF206
Tygon tube	Saint-Gobain K.K.	ACFJ00001
Tygon tube	Saint-Gobain K.K.	ACFJ00004
Microinjector	BEX CO., LTD.	BJ-120
Air blower	MonotaRO Co.,Ltd.	74003939
Arduino	Arduino	UNO R4 WiFi
Modulino Buttons	Arduino	Modulino Buttons
Optical fiber cannula	RWD Life Science Co.,LTD	R-FOC-BF400C-50NA
Charcoal powder	Taketora	Bamboo Charcoal Powder (15 micron)
LED driver	Thorlabs Inc.	LEDD1B
470 nm LED	Thorlabs Inc.	M470F3
405 nm LED	Thorlabs Inc.	M405FP1
Optical fiber cable to connect between optics	Thorlabs Inc.	M25L01
Fiber collimator for excitation cable	Thorlabs Inc.	F220SMA-A
Optical adapter for excitation cable fiber collimator	Thorlabs Inc.	KAD11F
Lens tube	Thorlabs Inc.	SM1L05
Filter cube	Thorlabs Inc.	DFM1/M
470 nm optical filter set	Thorlabs Inc.	MDF-GFP2
405 nm excitation filter	Thorlabs Inc.	FBH405-10
Dichroic mirror for 405 nm excitation	Thorlabs Inc.	DMLP425R
Post for filter cube	Thorlabs Inc.	RS1P/M
Clamp for filter cube	Thorlabs Inc.	CF125C
Connector for filter cube	Thorlabs Inc.	C4W-CC
End cap for filter cube	Thorlabs Inc.	SM1CP2
Adapter for patch cable fiber collimator	Thorlabs Inc.	AD15F
Fiber collimator for patch cable	Thorlabs Inc.	F950SMA-A
Lens for PMT	Thorlabs Inc.	LA1422-A-ML
Cage plate for PMT	Thorlabs Inc.	CP33/M
Cage plate for PMT	Thorlabs Inc.	DTCP1/M
Adapter for PMT	Thorlabs Inc.	DT108
Rod for cage assembly	Thorlabs Inc.	ER1
Breadboard	Thorlabs Inc.	MB2530/M
Photomultiplier Tube	Thorlabs Inc.	PMT1001/M
Optical fiber patch cable	Thorlabs Inc.	M126L01
Interconnect	Thorlabs Inc.	ADAF2
Multifunction I/O device	National Instruments Corp.	USB-6002
Camera	Teledyne Vision Solutions	CM3-U3-13Y3M-CS
Infrared illuminator kit	AKIZUKI DENSHI TSUSHO CO., LTD.	AE-LED56V2
Lens	Kowa Optronics Co.,Ltd.	LM5JC1M
Silicon probe	ATLAS Neuroengineering	E32+R-150-S2-L6-360NT
3D printer	Formlabs Inc.	Form 3+
Copper tape	Nitoms, Inc.	J3160
Pliers	KENOH Trading Co,.Ltd.	24022
Forceps	MODDIY	ESD-15
Epoxy adhesive	Konishi Co., Ltd.	16131
Cyanoacrylate adhesive	ALTECO co., ltd.	S01 PA
Resin for 3D print	Formlabs Inc.	RS-F2-GPCL-41
Pin header (17 mm length)	DMART (SHENZHEN) COMPANY LIMITED	40-Pin Single Row Male Pin Header, 2.54mm Pitch, 17mm Length
Long screw (M1.2, 14 mm length)	Matsumoto Sangyo Co., Ltd.	TD-00224
Nut (M1.2×3×1)	Soshin Industry Co., Ltd.	a3016501200000_2
Short screw (M1, 2 mm length)	SUNCO INDUSTRIES Co.,Ltd.	CSPPN1-SUS-M1-2
Spring scissors	Fine Science Tools Inc	15650–11
Micro-pointer	Fine Science Tools Inc	10066–15
Headstage	Intan Technologies	C3314
Commutator	Adafruit Industries	1196
Silicon-based adhesive	World Precision Instruments	KWIK-SIL
Rotary tool	Ginelson	YJP01
Benchtop fluorescence microscope	Evident Corporation	APX100

## Materials and equipment

### Meloxicam saline solution

Meloxicam saline solution (2 mg/mL) was prepared by adding 1 mL of meloxicam (20 mg/mL) into 9 mL of saline. The solution can be stored at room temperature for up to 3 months.

### Dexamethasone stock solution

Dissolve 100 mg of dexamethasone in 5 mL of dimethyl sulfoxide (DMSO) to prepare 5 mL of stock solution with a final dexamethasone concentration of 20 mg/mL. Store the stock solution at −20°C. The stock solution can be stored for at least six months under these conditions.

### Dexamethasone working solution

Before administration, prepare 1 mL of working solution by mixing 10 μL of dexamethasone stock solution (20 mg/mL) with 990 μL of saline, yielding final concentrations of 0.2 mg/mL dexamethasone and 1% (v/v) DMSO. Prepare the working solution immediately before use; do not store.

## Step-by-step method details

The overall experimental workflows and timelines for the independent EEG, fiber photometry, and silicon probe recording protocols are summarized in Figure 1.

**Figure 1:**
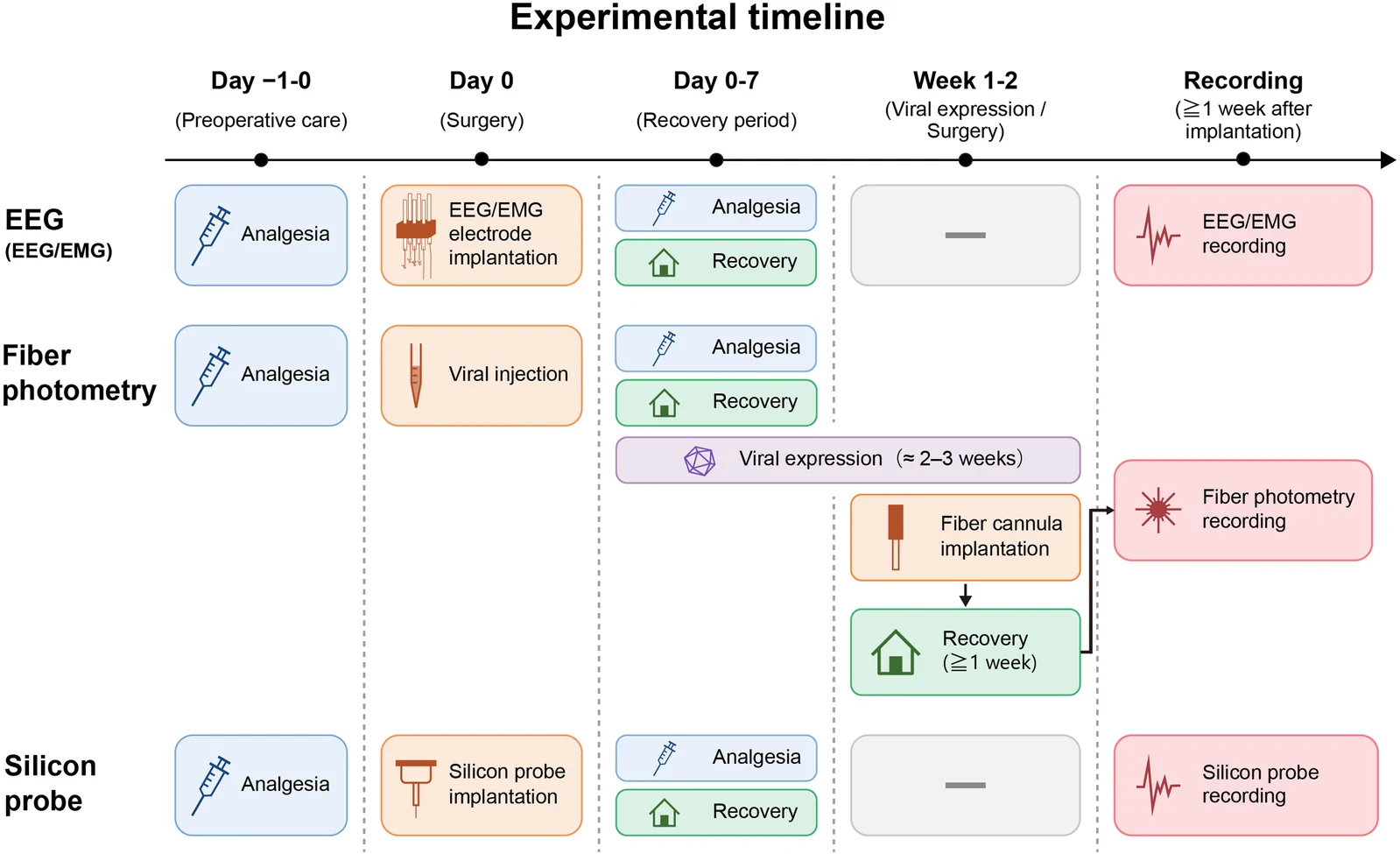
Experimental timeline for EEG, fiber photometry, and silicon probe recordings The timeline summarizes the experimental workflow for each recording modality, including analgesia, surgery, viral expression, recovery, and recording. EEG, fiber photometry, and silicon probe recordings represent independent experimental workflows and are not intended to be performed sequentially in the same animal.

### Habituation to handling

**Timing: Whenever applicable, preferably before recording sessions. Steps 1–3 are typically performed on consecutive days. Duration: 1 min/day/animal over multiple consecutive days.**

1. On the first day, gently stroke the animal’s back for 1 min to habituate it to handling.
2. On the second day, gently scoop the animal up with both hands and hold it for 1 min.

***Note:*** The animal may attempt to escape from your hands. If this occurs, continue gently scooping and handling the animal for the remainder of the 1-min session. If the animal shows a submissive posture with the ventral side exposed, return to Step 1.

3. Once the animal can be held stably in your hands, hold it in cupped hands while gently stroking its back for 1 min.

***Note:*** Once the animal is well habituated to handling, the effect may persist for more than 2 weeks.

***Note:*** Prior handling reduces struggling behavior during attachment of recording cables to the implanted EEG electrode, optical fiber cannula, or silicon probe during each recording session.

### Pre-surgical analgesia

**Timing: Between the day before and immediately before each surgery, duration: 5 min**

***Optional:*** Place the hamster in the anesthesia induction chamber and induce anesthesia with 3% isoflurane (flow rate: 0.5 L/min).

4. Measure the animal body weight to determine drug injection volume.
5. Administer the nonsteroidal anti-inflammatory drug meloxicam saline solution (1 mL/kg) subcutaneously using a 1-mL syringe.

***Note:*** Habituate animals to handling before surgery whenever possible. You can perform a subcutaneous injection without anesthesia when the animal is well habituated to handling.

### Skull exposure

**Timing: 0.5–1 h**

The following skull exposure procedure is common to all surgical protocols described below, including EEG electrode implantation, viral injection for fiber photometry recordings, optical fiber cannula implantation, and silicon probe implantation.

6. Prepare all surgical instruments (Figure 2A).

**Figure 2:**
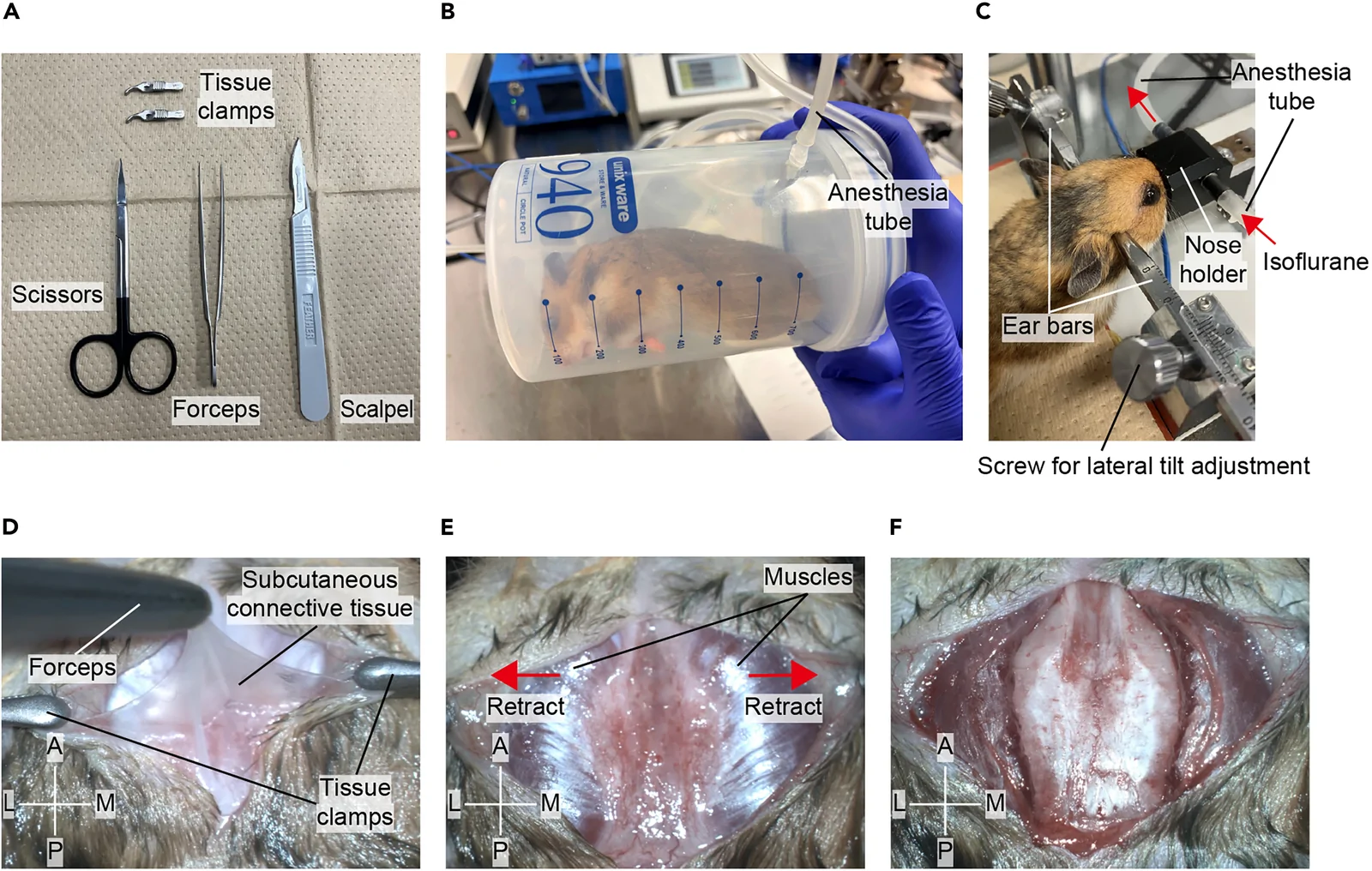
Surgical preparation and skull exposure in the Syrian hamster (A) Surgical instruments used for skull exposure. (B) A Syrian hamster placed in an anesthesia induction chamber. (C) A Syrian hamster secured in a stereotaxic apparatus. (D) Subcutaneous connective tissue covering the skull surface. (E) Muscles covering the skull after removal of the subcutaneous connective tissue. Red arrows indicate the direction of muscle retraction. (F) Skull surface exposed after muscle retraction.
7. Place the hamster in the anesthesia induction chamber and induce anesthesia with 3% isoflurane (flow rate: 0.5 L/min) (Figure 2B).

**CRITICAL:** Observe the animal’s respiration carefully. Syrian hamsters easily stop respiration by a high concentration of isoflurane exposure.

8. Once respiration stabilizes at approximately one breath per second, transfer the animal onto a heating pad placed on a stereotaxic apparatus, then secure the hamster’s head using two ear bars, an incisor bar, and a nose holder (Figure 2C).

***Optional:*** If the animal moves while secured in the ear bars, wrap the area around the mouth and the nose holder with loosely rolled Kimwipes to prevent isoflurane leakage.

9. Maintain isoflurane anesthesia at 3%.

***Note:*** During the following steps, if the animal breathes less than once every 3 s, decrease the isoflurane concentration gradually while carefully monitoring respiratory rate not to exceed 1 Hz.

10. Apply ophthalmic ointment to the eyes.

***Optional:*** Shave the fur on the scalp.

11. Disinfect the skin with 70% ethanol or iodine solution.

***Note:*** Use iodine solution only when you shave the fur on the scalp. Using iodine on the scalp without removing the fur may mat the fur and negatively affect the skin condition after surgery.

12. Make a longitudinal midline incision approximately 1.5–2 cm in length along the scalp.

***Note:*** From this point onward, if the hamster shows signs of pain and begins to awaken during surgery, see troubleshooting, problem 1.

***Note:*** If uncontrolled bleeding from the skull surface or muscle occurs during surgery, see troubleshooting, problem 3.

13. Remove subcutaneous connective tissue using scissors (Figure 2D).
14. Using a scalpel, detach the muscles covering the skull surface and retract them laterally (Figures 2E and 2F).

***Note:*** The region around the skull midline is covered by a thin membrane, which should also be carefully removed using a scalpel.

***Note:*** Unlike mice, Syrian hamsters possess relatively thick muscles covering the skull surface. The thickness and morphology of these muscles can vary substantially depending on age and individual differences.

**CRITICAL:** Care should be taken to detach and retract the muscles without cutting them whenever possible, as excessive damage may increase bleeding, reduce surgical stability, and affect post-surgical behavior after recovery.

### EEG electrode preparation

**Timing: Before electrode implantation surgery, duration: 0.5–1 h**

The following sections (Steps 15–33) describe the EEG recording protocol, including EEG electrode preparation (Steps 15–20), EEG electrode implantation surgery (Steps 21–31), and EEG recordings (Steps 32–33).

15. Prepare all required tools and materials (Figure 3A).

**Figure 3:**
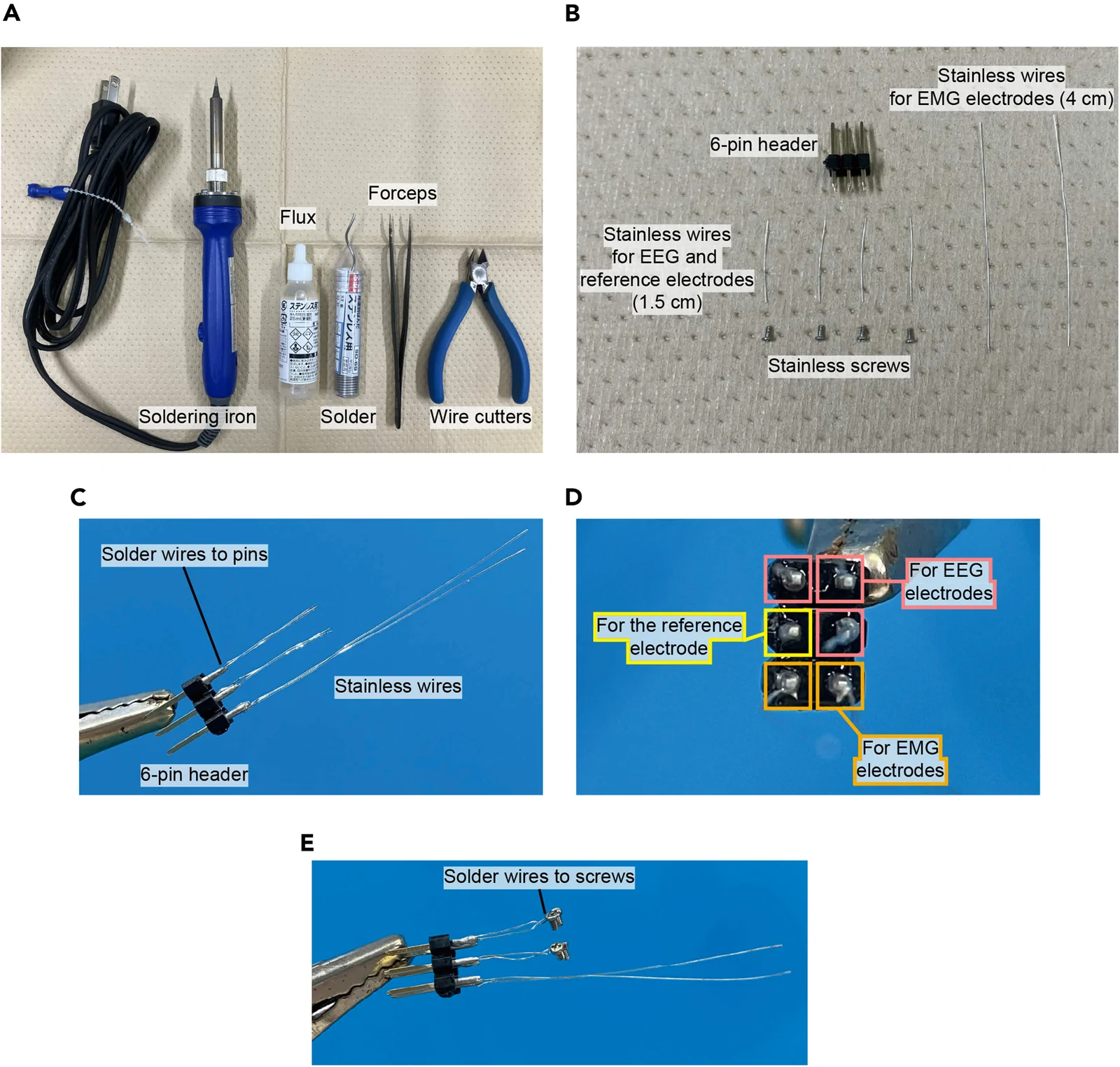
Preparation of EEG/EMG electrodes for chronic recordings (A) Tools used for EEG/EMG electrode preparation. (B) Components of the EEG/EMG electrode assembly, including a 6-pin header, stainless steel screws, and stainless steel wires for EEG/reference electrodes and EMG electrodes. (C) Stainless steel wires soldered to the pins of the 6-pin header. (D) Bottom view of the assembled EEG/EMG electrode configuration. Red, yellow, and orange boxes indicate the EEG, reference, and EMG electrodes, respectively. (E) Stainless steel screws soldered to the electrode wires for EEG and reference recordings.
16. Prepare a 6-pin header and insulated wires. Cut four 1.5-cm wires for EEG and reference electrodes, and two 4-cm wires for EMG recordings (Figure 3B).

***Note:*** The EMG wires are intentionally longer to improve maneuverability during surgery and to reduce the risk of displacement from the muscle during chronic recordings.

17. Remove approximately 1 mm of insulation from both ends of the EEG/reference wires and from one end of the EMG wires.

***Note:*** The insulation on the muscle-implantation side of the EMG wires should be removed immediately before implantation during surgery.

18. Solder the stripped wires to the short-side pins of the pin header (Figure 3C).

**CRITICAL:** Ensure that the solder attached to the short-side pins does not interfere with adjacent pins.

19. Attach stainless steel screws to the EEG and reference wires. Solder the wires to the underside of the screw heads (Figures 3D and 3E).

***Note:*** Because the screw electrodes are implanted into the skull, care should be taken to avoid excess solder on the screw tip or threads, as this may interfere with stable fixation into the skull.

***Note:*** Soldering can be facilitated by forming a small loop at the tip of the wire and hooking the loop onto the screw.

20. Spray 70% ethanol to remove residual flux and disinfect the electrode.

### EEG electrode implantation surgery

**Timing: 2–3 h**

21. Prepare all surgical instruments (Figure 4A).

**Figure 4:**
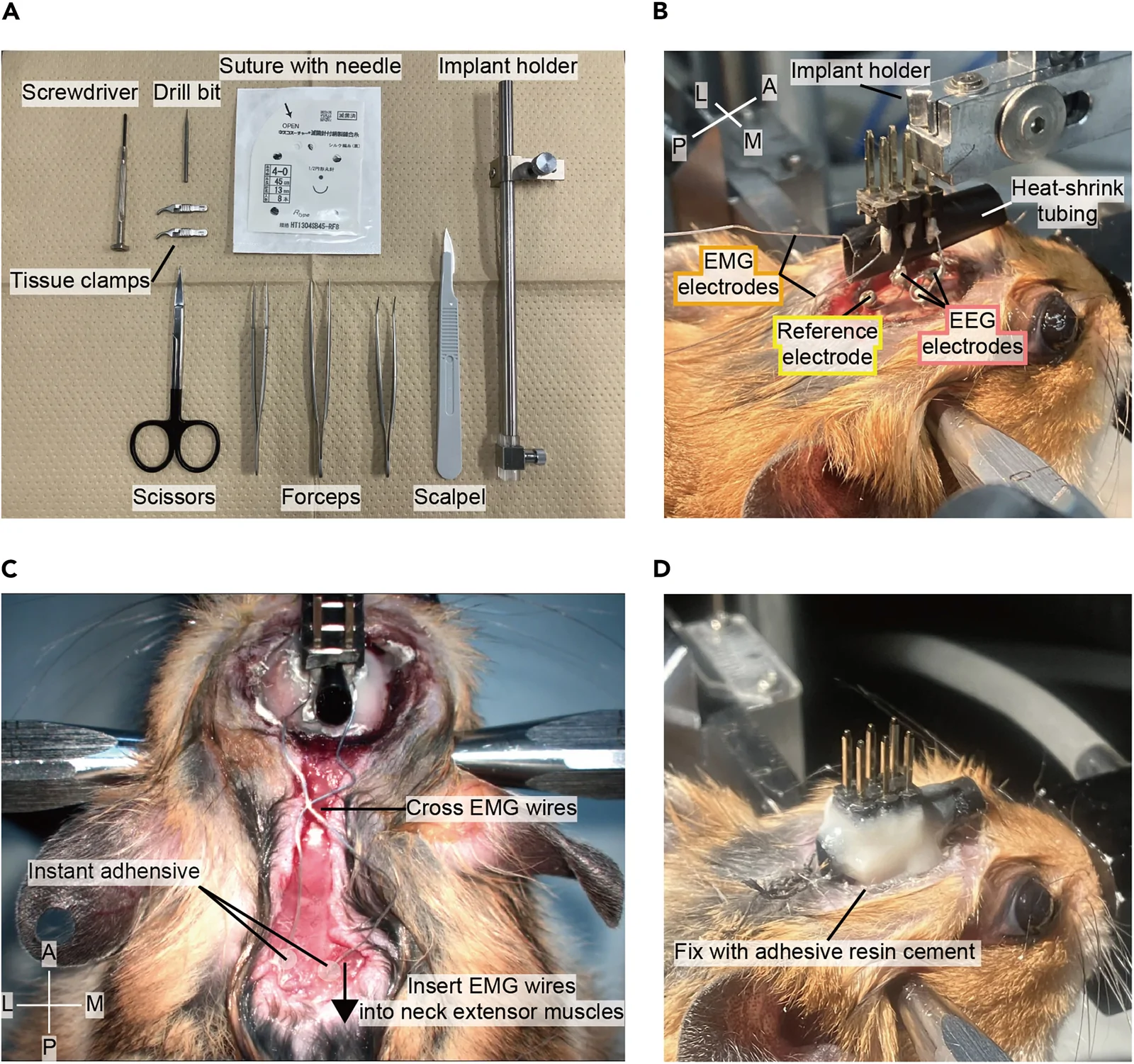
Implantation of EEG/EMG electrodes in the Syrian hamster (A) Surgical instruments used for EEG/EMG electrode implantation. (B) EEG, reference, and EMG electrodes positioned on the skull prior to fixation. Heat-shrink tubing was attached to improve connection stability during recordings. (C) EMG electrode implantation into the neck extensor muscles. The EMG wires were inserted in a crossed configuration and secured using instant adhesive. (D) Final fixation of the EEG/EMG electrode assembly using adhesive resin cement.
22. Anesthetize the hamster and perform the common skull exposure procedure (Figure 2).

***Note:*** From this point onward, if the hamster shows signs of pain and begins to awaken during surgery, see troubleshooting, problem 1.

***Note:*** If uncontrolled bleeding from the skull surface or muscle occurs during surgery, see troubleshooting, problem 3.

23. Perform skull etching.
  a. Apply enamel etching gel (Super-Bond C&B Red Activator) to the skull surface for 30 s.
  b. Rinse thoroughly with saline and wipe the surface with a cotton swab.
  c. Apply dentin etching solution (Super-Bond C&B Green Activator) for 5 s and repeat the same procedure described in b.

***Note:*** This treatment mildly decalcifies and roughens the bone surface, thereby improving adhesion of the resin cement. Excessive treatment, however, may weaken the skull surface; therefore, treatment duration should be carefully controlled.

24. Adjust the ear bars and incisor bar so that bregma and lambda are aligned on the same mediolateral (ML) and dorsoventral (DV) planes.

***Note:*** If bregma and/or lambda are difficult to identify, see troubleshooting, problem 2.

25. Drill four holes in the skull: one in the frontal region at AP +1.0 mm and ML −1.0 mm from bregma; two in the parietal region at AP −1.5 mm and ML ±2.0 mm from bregma; and one over the cerebellum for the reference electrode at AP −1.0 mm and ML −0.8 mm from lambda.

**CRITICAL:** Care should be taken not to fully penetrate the skull while drilling. The holes should be enlarged only until the diameter approximately matches that of the drill tip.

**CRITICAL:** Electrodes should not be placed directly on skull sutures, as fixation tends to become unstable along the suture lines.

26. Insert heat-shrink tubing between the pins of the pin header (Figure 4B).

***Note:*** Similar tubing should also be attached to the recording cable. During recordings, the two tubes can be tied with a wire to prevent accidental detachment of the recording cable from the electrode assembly.

27. Insert the screw electrodes into the drilled holes until the screw tips make gentle contact with the dura mater, while avoiding excessive pressure on the brain surface. Bend the wires beneath the pin header and position the pin header close to the skull surface (Figure 4B).

***Optional:*** If screw electrodes or wires are likely to contact each other, cover the contact points with dental adhesive resin (e.g., Transbond).

***Note:*** If Transbond adheres to the skin or muscle tissue during surgery, see troubleshooting, problem 4.

***Note:*** If the screw electrode becomes detached from or loose in the skull, see troubleshooting, problem 6.

***Note:*** If the wire twists during screw electrode implantation, making it difficult to manipulate the other wires and the pin header, see troubleshooting, problem 7.

28. Fix the skull, electrodes, and tubing using adhesive resin cement (Super-Bond).

***Note:*** Polymerization proceeds by mixing the monomer liquid (4-META) and polymer powder (PMMA) using tributylborane (TBB) as the initiator. The surface roughening procedure described in Step 23 improves bonding strength of the cement to the skull surface.

29. Make an additional 2–3 cm dorsal skin incision extending caudally from the head. Insert the two EMG wires into the neck extensor muscles in a crossed configuration and secure them with instant adhesive (Aron Alpha A “Sankyo”) (Figure 4C). Suture the incision using surgical sutures.

***Note:*** Crossing the wires helps prevent them from slipping out of the neck extensor muscles during chronic recordings.

***Note:*** If the EMG wire is difficult to insert into the neck extensor muscles, see troubleshooting, problem 8.

***Note:*** If the animal moves during EMG wire insertion, see troubleshooting, problem 9.

***Note:*** EMG wire implantation can be performed while waiting for the adhesive resin cement to harden.

30. Apply dental acrylic resin over the adhesive resin cement to secure the entire electrode assembly firmly to the skull (Figure 4D).

***Note:*** Insufficient contact between the skull and resin cement may result in implant detachment. See troubleshooting, problem 10.

***Note:*** If dental acrylic resin adheres to the skin or muscle tissue during surgery, see troubleshooting, problem 4.

31. Apply lidocaine and tetracycline antibiotic ointment (Terramycin) to the sutured region. Subsequently, administer the nonsteroidal anti-inflammatory drug meloxicam saline solution subcutaneously.

***Note:*** After surgery, check the implantation site daily for inflammatory responses, including pus formation, around the adhesive resin cement or dental acrylic resin. If such responses are observed, see troubleshooting, problem 5.

### EEG recordings

**Timing: After EEG electrode implantation surgery, allow at least 1 week for postoperative recovery before recording. The overall recording period is variable depending on the experimental design; in our experience, stable EEG recordings have been obtained for up to 3–4 months after implantation. Duration: Variable.**

Figure 5A shows a Syrian hamster after implantation of the EEG/EMG electrodes.

32. Assemble the EEG recording setup (Figure 5B).

***Note:*** A schematic illustration of the recording system is shown in Figure 5B. To prevent cable twisting during freely behaving recordings, the recording cable is connected through a commutator. Signals acquired from the electrodes are first amplified using a preamplifier and subsequently transmitted to the acquisition board (Open Ephys acquisition board). After analog-to-digital conversion, the signals are band-pass filtered and stored on a PC SSD. EEG/EMG signals are recorded relative to the reference electrode.

**Figure 5:**
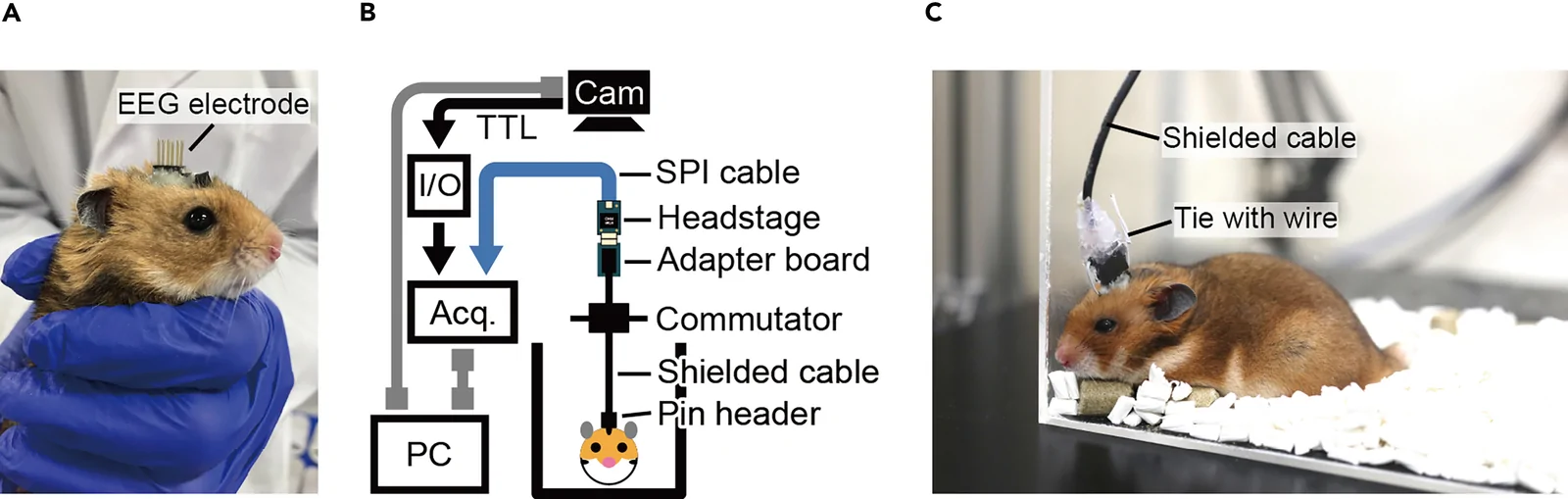
EEG recording system for freely moving Syrian hamsters (A) A Syrian hamster implanted with EEG/EMG electrodes. (B) Schematic diagram of the EEG/EMG recording system. Gray and black lines indicate USB and other cable connections, respectively. TTL, transistor-transistor logic; I/O, input/output; Acq., acquisition board. (C) A freely moving Syrian hamster connected to the shielded recording cable. The recording cable and electrode assembly were additionally secured using wire to prevent accidental detachment during recordings.

Behavioral activity of the hamster is simultaneously monitored using one or more cameras. Frame synchronization signals (TTL signals) generated by the cameras are input into auxiliary channels of the Open Ephys acquisition board and recorded on the same time axis as the electrophysiological signals. This configuration enables synchronization between EEG recordings and behavioral video recordings. Video acquisition and processing are performed using Bonsai, and timestamps for individual frames are saved as CSV files. By combining TTL-based synchronization signals with timestamp information acquired through Bonsai, temporal alignment between behavioral videos and electrophysiological recordings can be independently verified.

***Optional:*** You can monitor behavioral activity of the hamster during the dark phase using infrared lights in the recording system.

33. Connect the implanted electrodes to a 6-pin header connector attached to the animal’s head (Figure 5C), which is then connected to the recording system via a shielded 8-core cable.

**CRITICAL:** Ensure that there are no edges on the cage which may catch the cable during recording. If there are any such edges, cover them using masking tape.

***Optional:*** Tie the heat-shrink tubing on the implanted electrode assembly and the connector with a wire to improve connection stability and prevent accidental detachment during recordings.

***Note:*** If the electrode implant becomes detached during recording, see troubleshooting, problem 10.

### Viral injection

**Timing: Before optical fiber cannula implantation, duration: 3 h**

34. Prepare a glass pipette by pulling a glass capillary tube using a micropipette puller with the following instrument-defined arbitrary unit settings: Heat 784, Pull 10, Velocity 255, Delay 255, and cut the tip appropriately under a microscope to obtain the desired diameter.

***Note:*** Puller parameters may require adjustment depending on variability among filament batches.

***Note:*** You can prepare glass pipettes and store them before the surgery.

***Note:*** You can select glass capillary tubes according to the target volume of injection.

35. Add scale markings to the glass pipette to estimate injection volume (Figure 6A).

**Figure 6:**
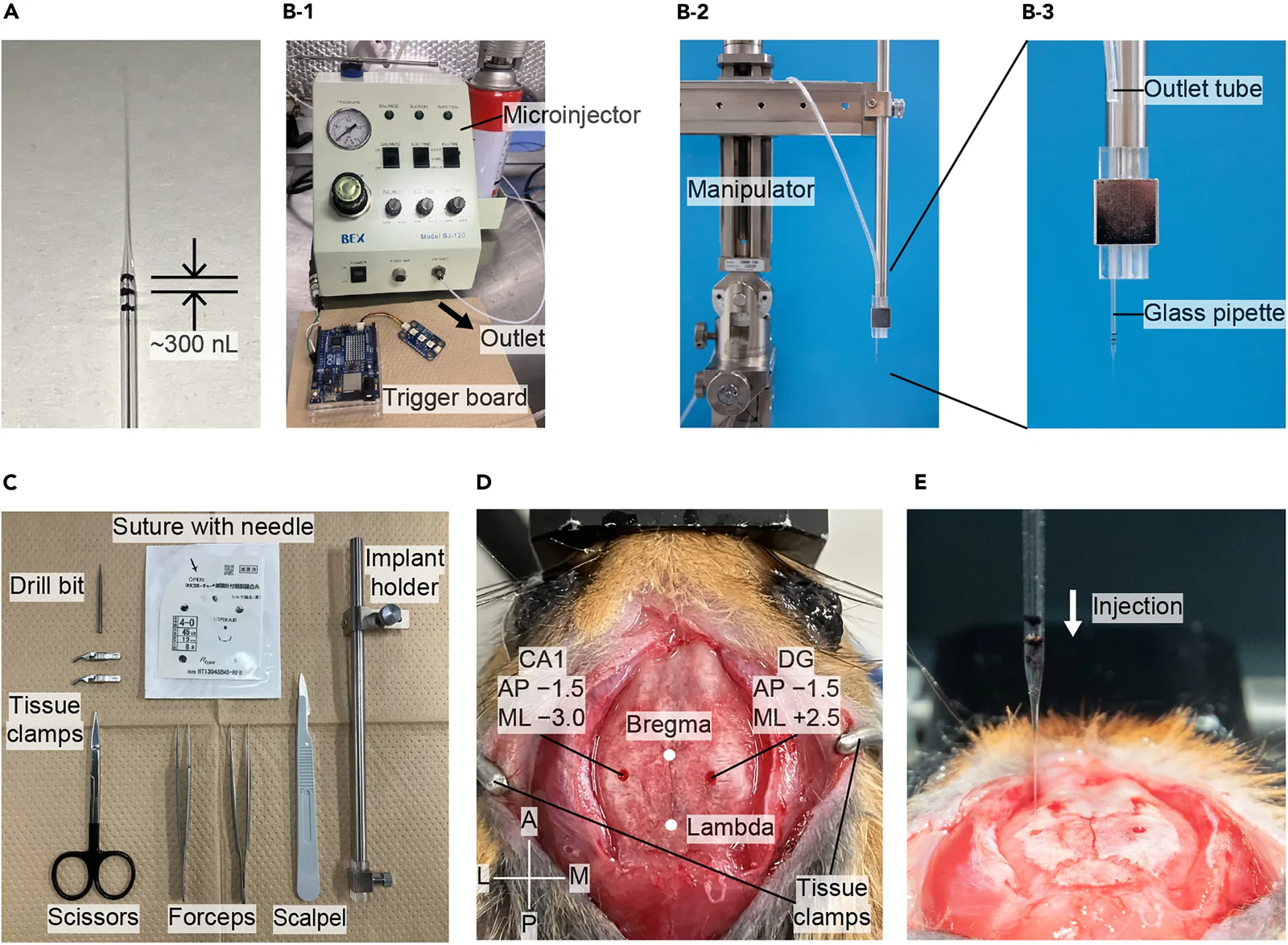
AAV injection procedure for fiber photometry recordings (A) Glass pipette marked for estimation of injection volume. (B) Microinjector system used for viral injection. (B-1) Microinjector equipped with a trigger board. Air pressure is delivered through the outlet tube. (B-2) Manipulator holding the glass pipette. (B-3) Enlarged view of the glass pipette connected to the outlet tube. (C) Surgical instruments used for viral injection. (D) Skull surface showing drilled holes for viral injection into the hippocampal CA1 and dentate gyrus (DG). (E) Viral injection performed using the glass pipette.

***Note:*** The scale markings are made based on the boundary between the pulled thin region and the original outer diameter of the capillary as a reference point, and additional marks corresponding to injection volume are added from this position.

36. Fill the glass pipette with viral solution by applying negative pressure with an attached 1-mL syringe without a needle via a plastic tubing.

***Note:*** Filling the pipette with an additional volume is recommended for test pumping.

37. Connect the glass pipette to an electric air-pressure microinjector (Figure 6B) and mount it onto the manipulator holder.
38. Prepare all surgical instruments (Figure 6C).
39. After completing the preparations described in Steps 34–38, anesthetize the hamster and perform the common skull exposure procedure (Figure 2).

***Note:*** From this point onward, if the hamster shows signs of pain and begins to awaken during surgery, see troubleshooting, problem 1.

***Note:*** If uncontrolled bleeding from the skull surface or muscle occurs during surgery, see troubleshooting, problem 3.

40. Adjust the ear bars and incisor bar so that bregma and lambda are aligned on the same ML and DV planes.

***Note:*** If bregma and/or lambda are difficult to identify, see troubleshooting, problem 2.

41. Mark the skull surface according to the stereotaxic coordinates of the injection target sites: CA1 at AP −1.5 mm and ML −3.0 mm from bregma, and the dentate gyrus (DG) at AP −1.5 mm and ML +2.5 mm from bregma.

***Note:*** The coordinates of the target sites depend on your study.

42. Drill a hole in the skull at the marked position (Figure 6D).

**CRITICAL:** You must keep the brain surface intact as much as possible to enable you to accurately specify the DV coordinates at the time of injection.

43. Re-adjust the ear bars and incisor bar to ensure that bregma and lambda remain aligned on the same ML and DV planes.

***Note:*** Pressing the drill against the skull may alter the relative height of bregma and lambda. Therefore, stereotaxic alignment should be rechecked after drilling.

***Note:*** If bregma and/or lambda are difficult to identify, see troubleshooting, problem 2.

44. Position the glass pipette above the injection site and lower it to the target depth: 1.8 mm below the brain surface for CA1 and 2.4 mm below the brain surface for DG.

***Note:*** Because skull thickness varies substantially among Syrian hamsters, setting DV coordinates relative to the brain surface provides more accurate targeting than using bregma-based DV coordinates alone.

45. Inject the viral solution using the electric microinjector (Figure 6E).

***Note:*** In this protocol, injections are performed with 1 Hz, 50 ms pulses at a flow rate of 1 nL/s using the scale markings described in Step 35 as a guide for injection volume.

***Note:*** Before lowering the pipette to the target depth, confirm that the pipette is not clogged by briefly ejecting a small amount of viral solution. Carefully remove any expelled viral solution from the pipette tip with a clean cotton swab before lowering the pipette. After injection and withdrawal, pipette patency can also be confirmed by checking whether solution can still be expelled from the pipette tip.

***Note:*** Viral injection volume and speed should be modified based on the viral vector and the targeted brain region.

46. Remove the glass pipette 5 min after completion of the injection.

***Note:*** To minimize viral backflow, the pipette should initially be withdrawn slowly. For further guidance on accurate viral delivery to the target brain region, see troubleshooting, problem 12.

47. Suture the incision.

***Note:*** In this protocol, optical fiber cannulas are implanted 1 week after viral injection. Alternatively, fiber cannulas may be implanted immediately following viral injection. Delayed implantation was adopted in this protocol to minimize leakage of viral solution outside the target region through the inserted fiber tract.

48. Apply lidocaine and tetracycline antibiotic ointment (Terramycin) to the sutured region. Subsequently, administer the nonsteroidal anti-inflammatory drug meloxicam saline solution subcutaneously.

**Pause point:** Following viral injection, animals can be maintained for 1 to several weeks before optical fiber cannula implantation.

### Optical fiber cannula implantation

**Timing: After viral injection, typically 1 week, duration: 3 h**

49. Prepare all surgical instruments (Figure 7A).

**Figure 7:**
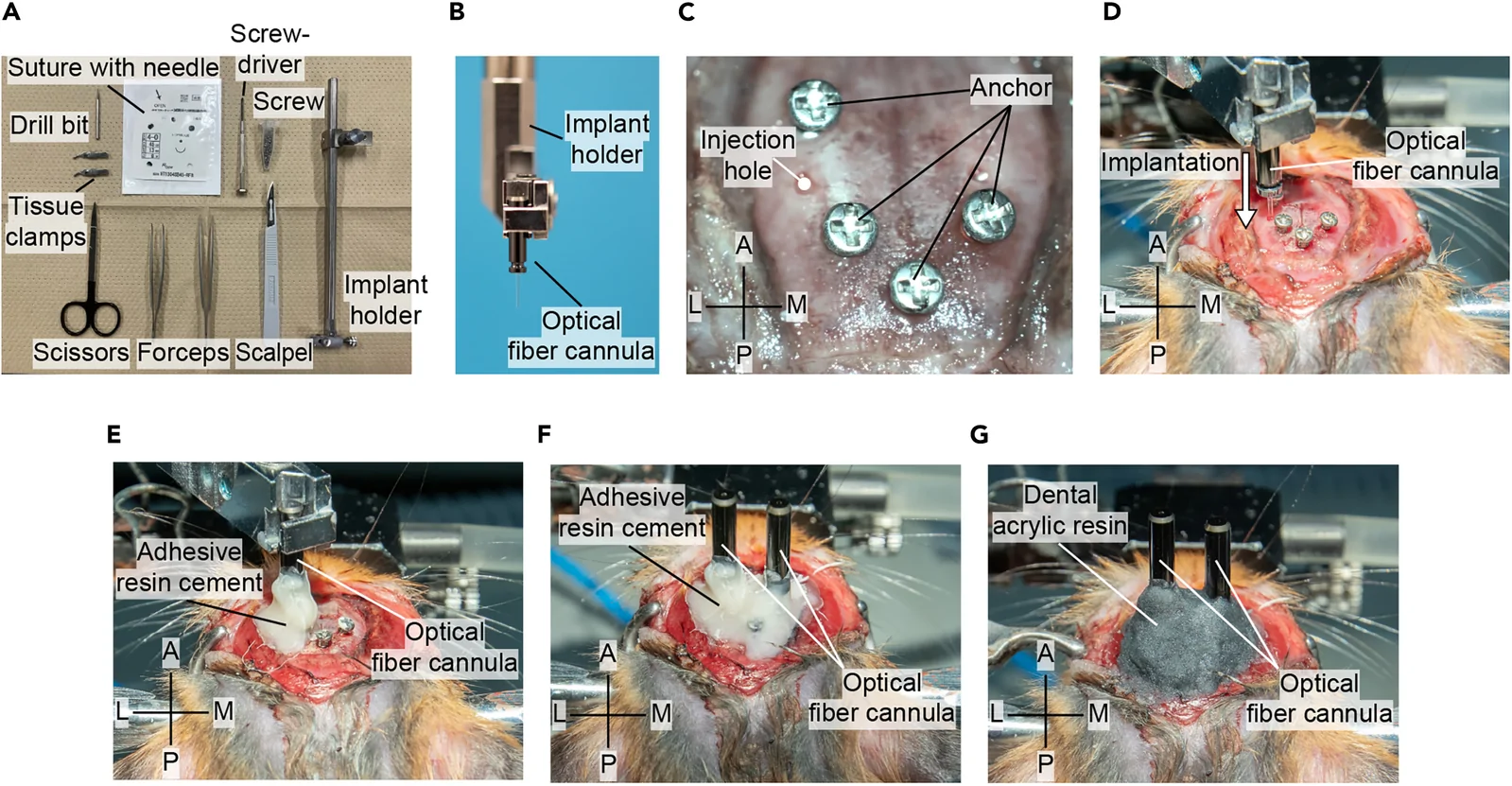
Optical fiber cannula implantation for fiber photometry recordings (A) Surgical instruments used for optical fiber cannula implantation. (B) Optical fiber cannula mounted on the implant holder. (C) Anchor screws implanted into the skull around the injection site. (D) Optical fiber cannula inserted into the brain. (E) Optical fiber cannula fixed to the skull using adhesive resin cement. (F) Simultaneous implantation of two optical fiber cannulas. (G) Adhesive resin cement covered with dental acrylic resin containing charcoal powder to reduce light leakage.
50. Anesthetize the hamster and perform the common skull exposure procedure (Figure 2).

***Note:*** From this point onward, if the hamster shows signs of pain and begins to awaken during surgery, see troubleshooting, problem 1.

***Note:*** If uncontrolled bleeding from the skull surface or muscle occurs during surgery, see troubleshooting, problem 3.

51. Perform skull etching as described in Step 23.
52. Mount the optical fiber cannula onto the holder (Figure 7B).

**CRITICAL:** Hold the cannula as close to the top as possible to keep the bottom surface for adhesive resin cement fixation.

53. Adjust the ear bars and incisor bar so that bregma and lambda are aligned on the same ML and DV planes.

***Note:*** If bregma and/or lambda are difficult to identify, see troubleshooting, problem 2.

54. Remove tissue covering the craniotomy site created during the viral injection surgery to expose the brain surface.

***Note:*** Approximately 1 week after viral injection, tissue may form over the craniotomy site and should be carefully removed when necessary. In addition, bleeding within the craniotomy site may contaminate the optical fiber cannula and introduce noise during recordings; therefore, the craniotomy site should be kept as clean as possible.

55. Drill four anchor holes and implant anchor screws into the skull (Figure 7C).

**CRITICAL:** Anchor fixation is unstable along skull sutures. Therefore, anchor screws should be placed at positions that do not interfere with the injection site and are located as far from the sutures as possible.

56. Re-adjust the ear bars and incisor bar to ensure that bregma and lambda remain aligned on the same ML and DV planes.

***Note:*** Pressing the drill against the skull may alter the relative height of bregma and lambda. Therefore, stereotaxic alignment should be rechecked after drilling.

***Note:*** If bregma and/or lambda are difficult to identify, see troubleshooting, problem 2.

57. Insert the optical fiber cannula into the brain (Figure 7D).

***Note:*** The implantation depth depends on the numerical aperture of the optical fiber cannula and the expression range of the fluorescent probe. In this protocol, the cannula is implanted 0.2 mm shallower than the viral injection depth.

58. Fix the cannula, anchor screws, and skull together using adhesive resin cement (Super-Bond) (Figure 7E).

**CRITICAL:** The top 2/3 of the cannula should be kept intact to properly attach a fiber connector at the time of recording. Be careful not to place the resin onto the upper side of the cannula.

59. After the cement hardens, detach the cannula from the holder.

***Note:*** When implanting multiple optical fiber cannulas, repeat Steps 52 and 56–59 for each cannula (Figure 7F).

60. Cover the adhesive resin cement with dental acrylic resin mixed with charcoal powder (Figure 7G).

***Note:*** Because adhesive resin cement is white, excitation light may scatter within the cement and leak outside the implant. Covering the implant with black dental acrylic resin containing charcoal powder helps suppress light leakage.

***Note:*** If dental acrylic resin adheres to the skin or muscle tissue during surgery, see troubleshooting, problem 4.

***Note:*** During surgery, secure the optical fiber cannula using a sufficient amount of cement over an adequate fixation area to prevent postoperative loosening. For further guidance, including considerations for postoperative housing, see troubleshooting, problem 13.

61. Moisten the skin surrounding the incision with saline to soften the tissue.
62. Suture the incision.
63. Apply lidocaine and tetracycline antibiotic ointment (Terramycin) to the sutured region. Subsequently, administer the nonsteroidal anti-inflammatory drug meloxicam saline solution subcutaneously.

***Note:*** After surgery, check the implantation site daily for inflammatory responses, including pus formation, around the adhesive resin cement or dental acrylic resin. If such responses are observed, see troubleshooting, problem 5.

### Fiber photometry recordings

**Timing: Begin fiber photometry recordings after sufficient expression of the viral construct has been achieved. The required expression period depends on the viral vector, promoter, and fluorescent sensor used. In our experiments, recordings were typically initiated 2–3 weeks after viral injection, followed by at least 1 week of recovery after optical fiber cannula implantation. Duration: Variable.**

Figure 8A shows a Syrian hamster after implantation of the optical fiber cannula.

64. Assemble the fiber photometry setup (Figure 8B).

***Note:*** A schematic illustration of the recording system is shown in Figure 8B. In this setup, GFP-based fluorescent sensors, including GCaMP and GRAB sensors, are excited using a 470 nm LED through a filter set containing excitation filters and dichroic mirrors. For acquisition of reference (isosbestic) signals, a 405 nm LED is used as an additional excitation light source through a separate excitation filter and dichroic mirror.

**Figure 8:**
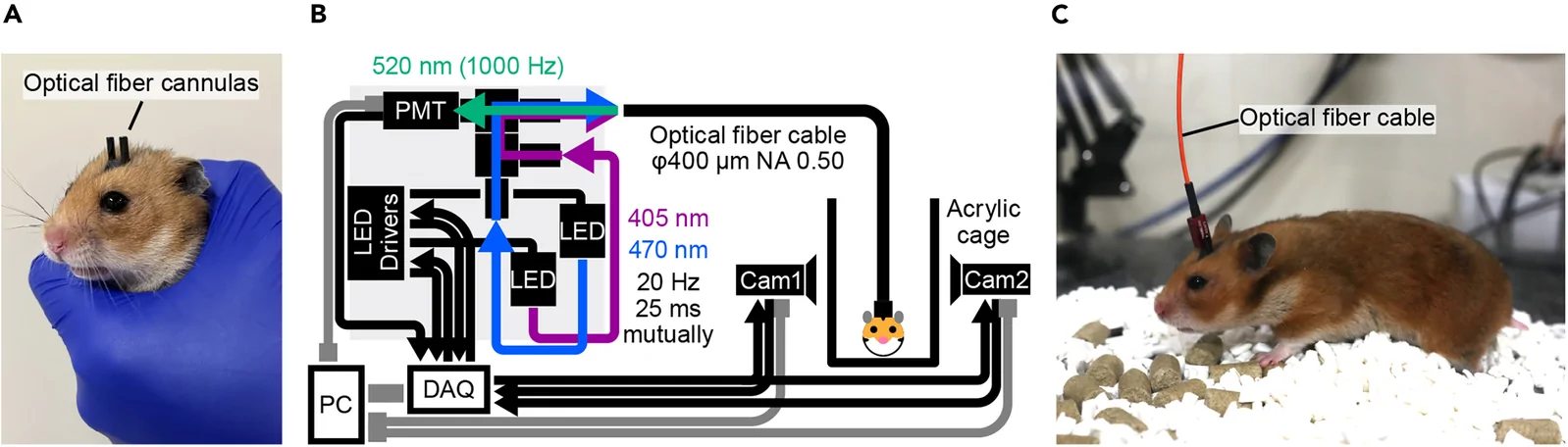
Fiber photometry recording system for freely moving Syrian hamsters (A) A Syrian hamster implanted with optical fiber cannulas. (B) Schematic diagram of the fiber photometry recording system. Gray and black lines indicate USB and other cable connections, respectively. PMT, photomultiplier tube; DAQ, data acquisition board. (C) A freely behaving Syrian hamster connected to the optical fiber cable during recordings.

Excitation light is delivered to the brain through the optical fiber cannula via an optical pathway consisting of optical fiber cables, collimators, filter cubes, and a 400-μm core diameter optical fiber cable (numerical aperture: 0.5). Emitted fluorescence propagates backward through the same optical path and is detected by a photomultiplier tube (PMT) through the filter cube containing fluorescence filters and collection optics.

LED intensity is controlled electronically, and PMT signals are acquired at 1 kHz using a multifunction I/O device and a custom LabVIEW program before being stored on a PC HDD. The 470 nm and 405 nm LEDs are alternately illuminated at 40 Hz. Light intensity for each LED is adjusted such that fluorescence signals evoked by 470 nm and 405 nm excitation are approximately −1 V and −0.5 V, respectively. These signal levels are optimized through combinations of PMT gain adjustment within PMT 2100 Control software, and excitation LED intensity adjustment within LabVIEW.

Animal behavior is simultaneously recorded using one or more cameras. Photometry signals and frame synchronization signals (TTL signals) generated by the cameras are simultaneously acquired on the same computer using LabVIEW. Video acquisition and processing are performed using Bonsai, and timestamps for each frame are saved as CSV files. Combining TTL synchronization signals with timestamp information acquired through Bonsai enables independent verification of temporal alignment between behavioral videos and fluorescence recordings.

65. Connect the optical fiber cable to the implanted optical fiber cannula with an interconnect (Figure 8C).

**CRITICAL:** Ensure that there are no edges on the cage which may catch the cable. If there are some edges, cover them using masking tape.

***Note:*** If fluorescence signals from the fluorescent sensor are weak or undetectable during recording, see troubleshooting, problem 11.

### Silicon probe microdrive assembly

**Timing: By the day before the implantation surgery, duration: 1 day and a half day**

The following sections (Steps 66–108) describe the silicon probe recording protocol, including silicon probe microdrive assembly (Steps 66–78), silicon probe implantation (Steps 79–100), silicon probe recordings (Steps 101–102), and silicon probe recovery (Steps 103–108).

The procedure for the microdrive assembly is visually demonstrated in the Methods Video S1.

Methods video S1. Microdrive assembly for silicon probe implantation, related to Steps 69–72, 74, 75 and 90 This video provides a visual overview of the microdrive assembly procedure described in Steps 69–72, 74, 75 and 90.

In this protocol, a silicon probe manufactured by ATLAS Neuroengineering (E32+R-150-S2-L6-360NT) was used.

66. Prepare all required tools and materials (Figure 9A).

**Figure 9:**
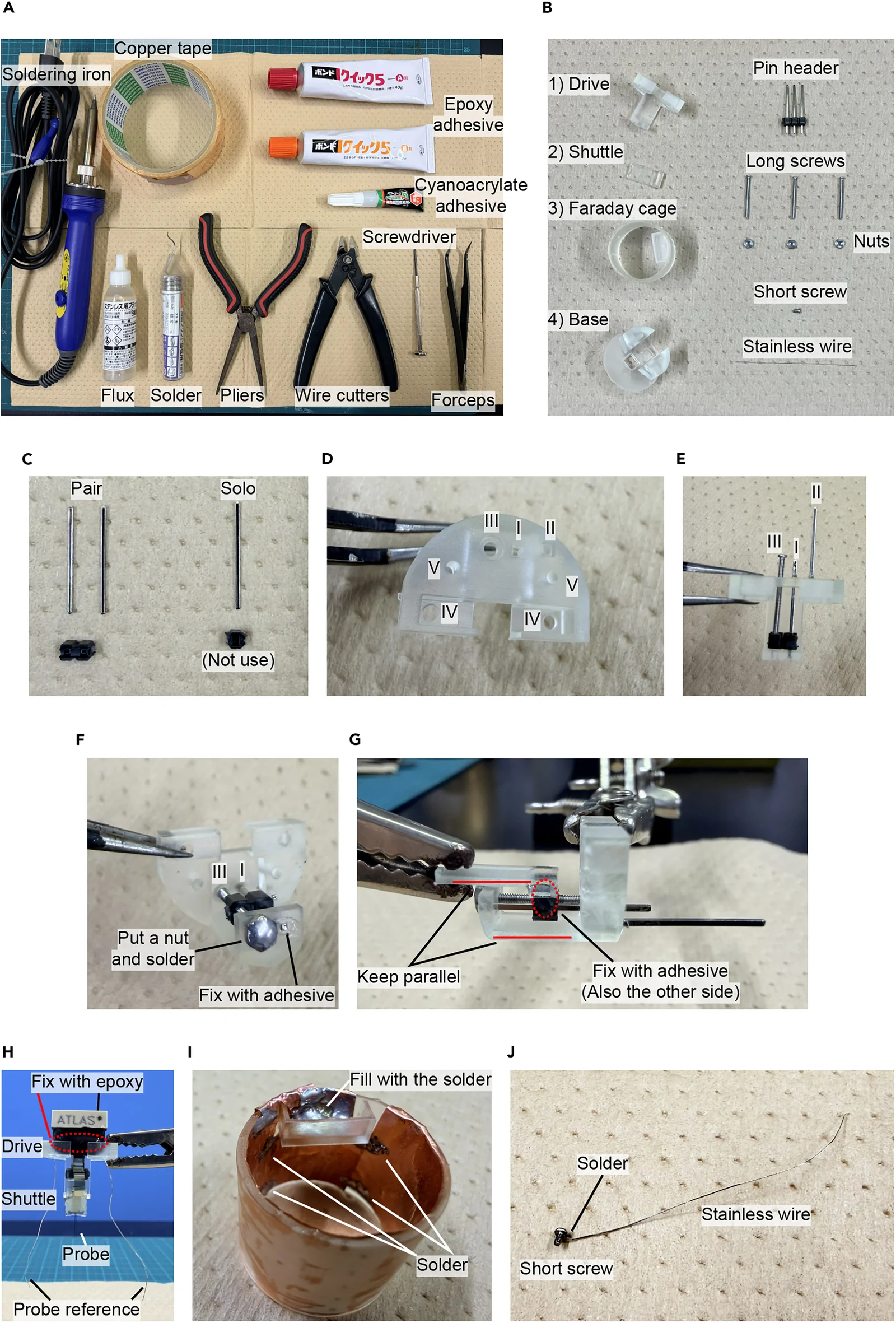
Assembly of the microdrive for chronic silicon probe recordings (A) Tools required for microdrive assembly. (B) Components used for microdrive assembly, including the drive body, shuttle, Faraday cage, and base. (C) Pin headers separated into paired and single pins. (D) Top view of the drive body showing holes and pockets used for assembly. (E) Assembly of the drive body with the long screw, pins, and housing. (F) Bottom view of the drive body after fixation of the nut. (G) Shuttle attached to the drive body while maintaining parallel alignment. (H) Silicon probe attached to the shuttle and drive assembly. (I) Inner surface of the Faraday cage coated with conductive copper tape. (J) Reference electrode prepared by soldering a stainless steel wire to a short screw.
67. Prepare the following components: parts 1–4, a 3-pin header, three screws, three nuts, one short screw, and a 4-cm wire (Figure 9B). Parts 1–4 correspond to the drive body, shuttle, Faraday cage, and base, respectively. These components were fabricated using a stereolithography (SLA) 3D printer based on the STL files deposited in Mendeley Data.

**CRITICAL:** During 3D printing, avoid placing support structures on surfaces where the parts will contact each other. Residual support material may interfere with proper fitting and smooth movement of the assembled components.

***Note:*** If you use a silicon probe with a different shank length, adjust the height of the base and drive shaft according to the difference in shank length and prepare the screws and pin headers according to changes in the length.

68. Separate the 3-pin header into a 2-pin header and a 1-pin header, and then separate the pins from the plastic housing (Figure 9C).

***Note:*** You can easily separate the pins from the housings using pliers.

69. Insert one pin into hole II of the drive body (Figure 9D) such that it does not fully penetrate the backside of the drive body, and secure it using instant adhesive (Figure 9E). This pin is later used to attach the microdrive to the stereotaxic implant holder during implantation and probe recovery.
70. Place the housing on the drive body so that it aligns with holes I and III of the drive body as shown in Figure 9E.
71. Insert one pin through the upper side of hole I in the drive body (Figure 9E), through the hole in the housing, and through the bottom hole of the drive body directly below hole I.
72. Insert a screw into hole III of the drive body (Figure 9D) such that it passes through the remaining hole of the housing installed in Step 70 (Figure 9E).
73. Secure the pin which was inserted into the drive body in Step 71 to the drive body using instant adhesive on the surface and the bottom of the drive body, followed by epoxy adhesive, while preventing it from flowing out toward the housing to ensure that the housing remains movable around the pin.
74. Attach a nut to the tip of the screw inserted in Step 72 and secure it by soldering (Figure 9F).

***Note:*** In this structure, one pin of the 2-pin header is replaced with a screw. The pin and screw are fixed to the drive body to prevent vertical movement while still allowing rotational movement of the screw. This design converts rotational motion of the screw into vertical linear movement of the housing. By fixing the shuttle component to the housing in subsequent steps, the insertion depth of the silicon probe can be precisely controlled according to the degree of screw rotation.

**CRITICAL:** After tightening the nut, loosen it by approximately 60° before fixation. Excessive tightening prevents smooth screw rotation, causing rotational force to be directly transmitted to the backside of the drive body during adjustment, which may damage the structure.

75. Attach the shuttle (Figure 9B-2) to the housing mounted on the drive body (Figure 9B-1) using instant adhesive, as shown in Figure 9G.

***Note:*** Before Step 76, you should check whether the shuttle moves up and down without displacing the pin by turning the screw in hole III in Figure 9D.

***Note:*** Turning the screw anticlockwise lowers the shuttle along the DV axis, whereas turning it clockwise raises it.

**CRITICAL:** Because the silicon probe will later be aligned along the shuttle (Figure 9B-2), the shuttle should be attached such that it is parallel to the axis of the drive body (Figure 9B-1). The precision of this alignment directly affects the stability of probe insertion and the stereotaxic accuracy of the recording site.

76. Insert the silicon probe through pocket IV of the drive body (Figure 9D), and fix the probe base to the shuttle (Figure 9B-2) using epoxy adhesive. Additionally, secure the silicon probe within pocket IV (Figure 9D) using epoxy adhesive. Subsequently, insert two electrode wires into the holes within pocket IV (Figure 9H).

***Note:*** Since the epoxy adhesive takes much time to fully cure, the assembled drive body should be left overnight.

**CRITICAL:** As in Step 75, care should be taken to ensure that the axis of the drive body (Figure 9B-1), the shuttle (Figure 9B-2), and the probe shank remain parallel during probe fixation. The precision of this alignment directly affects the stability of probe insertion and the stereotaxic accuracy of the recording site.

***Note:*** The epoxy adhesive may deform slightly during curing, resulting in probe misalignment. For guidance on preventing this issue, see troubleshooting, problem 14.

77. Apply conductive tape (copper tape) to the inner surface and pocket region of the Faraday cage (Figure 9B-3), and secure the junctions by soldering (Figure 9I).

***Note:*** Depending on the recording environment, the inside of the Faraday cage can become rusty. This rust prevents the smooth recovery of the silicon probe, a process shown in Figure 12. To avoid this, you should drill four small holes in the Faraday cage at 90° intervals.

78. Solder a screw and a wire together to prepare the reference electrode (Figure 9J).

***Note:*** Because the screw electrode will be implanted into the skull using a screwdriver, care should be taken to avoid excess solder on the screw tip or threads.

***Note:*** When using a reused probe, only the Faraday cage (Figure 9B-3), the base (Figure 9B-4), two long screws, two nuts, one short screw, and a stainless wire are required. Steps 66–76 can be omitted.

### Silicon probe implantation

**Timing: 3–6 h**

79. Prepare all surgical instruments (Figure 10A).

**Figure 10:**
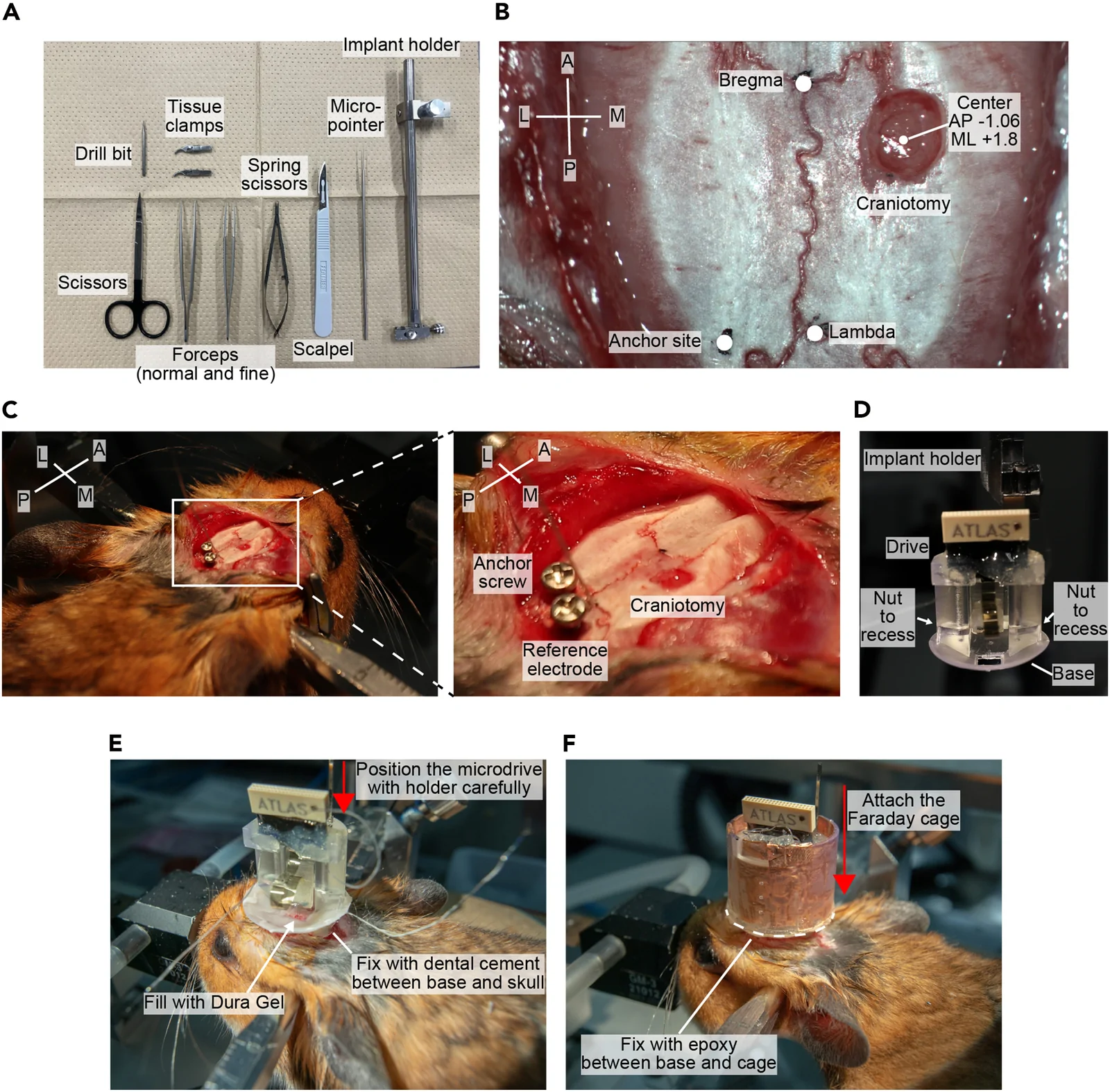
Surgical procedures for chronic silicon probe implantation (A) Surgical instruments used for silicon probe implantation. (B) Skull surface after craniotomy for hippocampal silicon probe implantation. The center of the craniotomy was positioned at AP −1.06 mm and ML +1.80 mm from bregma. (C) A head-fixed Syrian hamster after implantation of the anchor screws and reference electrode. The right panel shows an enlarged view of the region outlined by the white square in the left panel. (D) Fully assembled microdrive mounted on the implant holder. (E) Placement and fixation of the microdrive onto the skull surface. Dura Gel was applied to protect the exposed brain surface. (F) Final appearance of the implanted microdrive after attachment of the Faraday cage.
80. Anesthetize the hamster.
81. Administer dexamethasone solution intramuscularly into the hindlimb (0.2 mg/kg).

***Note:*** Compared with the other surgical procedures described in this protocol, silicon probe implantation requires a larger dural opening and insertion of the probe into the brain tissue, both of which may induce local inflammatory responses and increased vascular permeability. Dexamethasone is therefore administered preoperatively to suppress brain edema through its anti-inflammatory effects and ability to reduce vascular permeability.

82. Perform the common skull exposure procedure (Figure 2).

***Note:*** From this point onward, if the hamster shows signs of pain and begins to awaken during surgery, see troubleshooting, problem 1.

***Note:*** If uncontrolled bleeding from the skull surface or muscle occurs during surgery, see troubleshooting, problem 3.

83. Adjust the ear bars and incisor bar so that bregma and lambda are aligned on the same mediolateral (ML) and dorsoventral (DV) planes.

***Note:*** If bregma and/or lambda are difficult to identify, see troubleshooting, problem 2.

84. Identify the stereotaxic coordinates for silicon probe implantation in the right dorsal hippocampus at AP −1.06 mm and ML +1.80 mm from bregma.

***Note:*** These coordinates are for hippocampal recordings. The coordinates of the target sites depend on your study.

85. Thin the skull gradually using a drill over an area extending ±0.5 mm in both the AP and ML directions centered on the implantation coordinates. Once cracks appear in the thinned skull region, carefully remove bone fragments using forceps (Figure 10B).

***Note:*** The size of the craniotomy should be adjusted depending on the shaft pitch of the silicon probe used. Ideally, it should be at least twice as wide as the shaft pitch because there may be large blood vessels within the craniotomy.

86. Remove the dura mater within the craniotomy site using a micro-pointer or forceps.

***Note:*** First, make an incision with a micro-pointer, choosing an area with few blood vessels. Then, carefully expand the incision using forceps and/or a micro-pointer to enlarge the exposed area of the brain surface.

***Note:*** If any part of the excised dura mater remains attached to the edge of the craniotomy window, it may obstruct probe insertion. In this case, grasp the dural remnant with fine forceps and cut it away using spring scissors.

**CRITICAL:** Because the dura mater of Syrian hamsters is relatively tough, probe insertion is difficult unless the dura is removed.

**CRITICAL:** After dura removal, the brain surface becomes exposed and should be kept moist with saline to prevent drying. In addition, blood contamination on the probe may interfere with vertical movement after implantation and introduce recording noise. Therefore, sufficient hemostasis and maintenance of a clean surgical field are essential.

87. Implant the reference electrode and anchor screws using a precision screwdriver (Figure 10C).

***Note:*** Temporarily position the base beforehand to ensure that the electrodes, anchor screws, and base do not interfere with one another.

88. Re-adjust the ear bars and incisor bar so that bregma and lambda remain aligned on the same ML and DV planes.

***Note:*** If bregma and/or lambda are difficult to identify, see troubleshooting, problem 2.

89. Coat the silicon probe with the fluorescent dye DiI.

***Note:*** This procedure is performed to verify the probe insertion track during subsequent histological analyses. DiI solution should be handled using a toothpick or pipette tip; however, these tools should not directly contact the probe. Instead, apply the dye by bringing the liquid surface into contact with the probe tip. Direct contact may damage the probe.

90. Insert screws into hole V of the drive body (Figure 9D), position nuts within the recesses on both sides of the base (Figure 9B-4), and assemble the microdrive (Figure 10D).
91. Position the microdrive on the skull surface (Figure 10E).

***Note:*** When positioning the microdrive, ensure that the 3D-printed base does not interfere with the underlying muscle. If interference occurs, adjust the orientation of the base. If the problem persists, see troubleshooting, problem 15.

***Note:*** In addition to the dexamethasone administration described in Step 81, the osmotic agent glyceol is intraperitoneally administered 15 min before this procedure (15 μL/g) to reduce intracranial pressure.^4^

92. Lower the shuttle and adjust the probe height so that the probe tip contacts the brain surface.

**CRITICAL:** The probe should be placed away from large blood vessels to prevent excessive bleeding.

93. Fix the microdrive to the skull using adhesive resin cement (Super-Bond). After the cement has sufficiently hardened, turn the microdrive screw counterclockwise to lower the probe into the brain to the desired recording depth.

***Note:*** Care should be taken to prevent adhesive resin cement from contacting the probe, as this may introduce recording noise. In particular, avoid leakage of the cement into the craniotomy area.

94. Apply dura protection gel (Dura-Gel) over the craniotomy area.

***Note:*** Dura-Gel is a biocompatible soft silicone gel that functions as an artificial dura substitute. The gel is prepared by mixing two polymer components immediately before application.

***Note:*** Before applying the Dura-Gel, all recording channels on the probe should be in the brain to prevent it from adhering to the recording channels and affecting the recording quality.

**CRITICAL:** Confirm that the entire bottom surface of the base is firmly fixed with adhesive resin cement before applying Dura-Gel. If fixation is incomplete, the gel may leak into the boundary between the base and skull surface, resulting in insufficient fixation of the implant.

95. Reinforce fixation between the base (Figure 9B-4) and the skull using dental acrylic resin.

***Note:*** At a minimum, the outer surface of the base flange should be covered with dental acrylic resin to protect it from damage.

***Note:*** If dental acrylic resin adheres to the skin or muscle tissue during surgery, see troubleshooting, problem 4.

96. Attach the Faraday cage (Figure 9B-3) to the microdrive assembly (Figure 10F).

**CRITICAL:** The reference wires from both the reference electrode on the skull and the probe should be in the Faraday cage to prevent cutting the wires.

97. Within the pocket region of the Faraday cage (Figure 9B-3), solder the reference electrode wire to the silicon probe electrode wire. Subsequently, cover the soldered region with epoxy adhesive to electrically insulate it from the surroundings.
98. Bond the base (Figure 9B-4) and the Faraday cage (Figure 9B-3) together using epoxy adhesive.
99. Suture the skin incision.

***Note:*** Suturing is performed only when wound opening remains around the incision margins.

100. Apply lidocaine and tetracycline antibiotic ointment (Terramycin) to the sutured region. Subsequently, administer the nonsteroidal anti-inflammatory drug meloxicam saline solution subcutaneously.

***Note:*** After surgery, check the implantation site daily for inflammatory responses, including pus formation, around the adhesive resin cement or dental acrylic resin. If such responses are observed, see troubleshooting, problem 5.

### Silicon probe recordings

**Timing: After silicon probe implantation surgery, allow at least 1 week for postoperative recovery before recording. The overall recording period is variable depending on the experimental design; in our experience, stable recordings have been maintained for more than 1 month after implantation. Duration: Variable**

Figure 11A shows a Syrian hamster after implantation of the silicon probe.

101. Assemble the silicon probe recording setup (Figure 11B).

***Note:*** Procedures following signal transmission from the headstage to the acquisition board (Open Ephys acquisition board), as well as synchronization with behavioral video recordings, are performed in the same manner as described for EEG recordings (Figure 5).

102. Connect the silicon probe implanted in the head of the Syrian hamster to a headstage containing a built-in preamplifier (Figure 11C).

**CRITICAL:** Ensure that there are no edges on the cage which can catch the cable. If there are some edges, cover them using masking tape.

***Optional:*** For long-term recording, secure the connector junction with a silicone-based adhesive (Kwik-Sil) to prevent the SPI cable from becoming disconnected from the headstage. See troubleshooting, problem 16.

**Figure 11:**
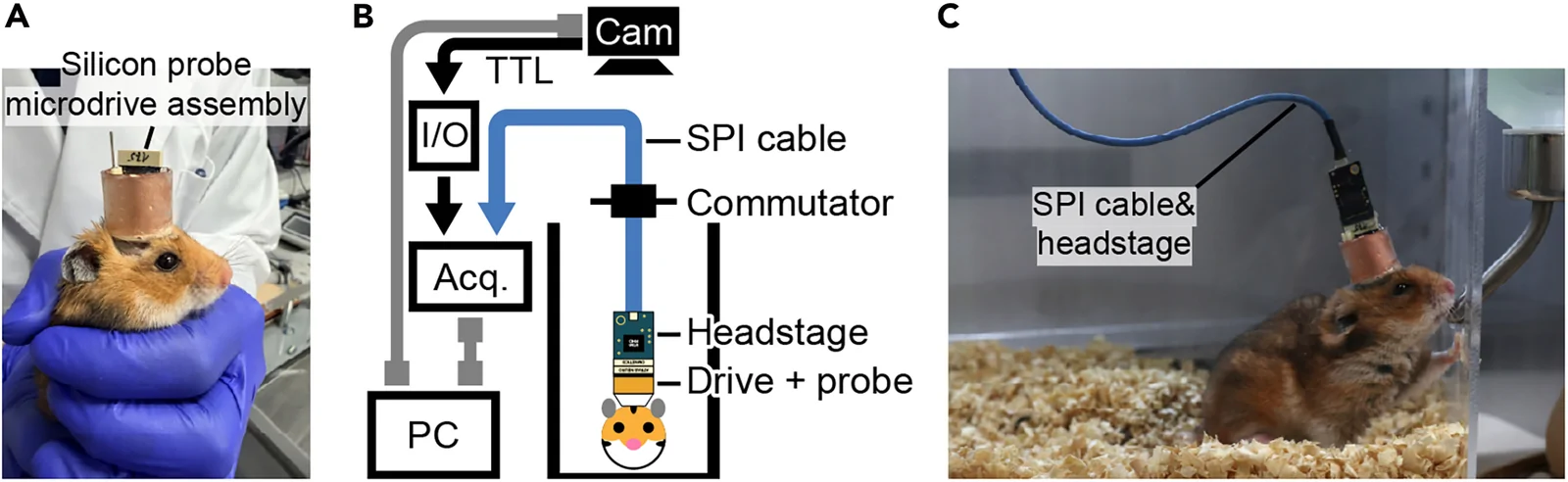
Silicon probe recording system for freely moving Syrian hamsters (A) A Syrian hamster implanted with a silicon probe microdrive assembly. (B) Schematic diagram of the silicon probe recording system. Gray and black lines indicate USB and other cable connections, respectively. TTL, transistor-transistor logic; I/O, input/output; Acq., acquisition board. (C) A freely behaving Syrian hamster connected to the SPI cable and headstage during silicon probe recordings.

### Silicon probe recovery

**Timing: After completing the recording, under deep anesthesia and before transcardial perfusion, duration: 30 min**

***Note:*** Silicon probes are recovered before transcardial perfusion to prevent paraformaldehyde contamination and enable probe reuse.

103. Prepare all required tools (Figure 12A).

**Figure 12:**
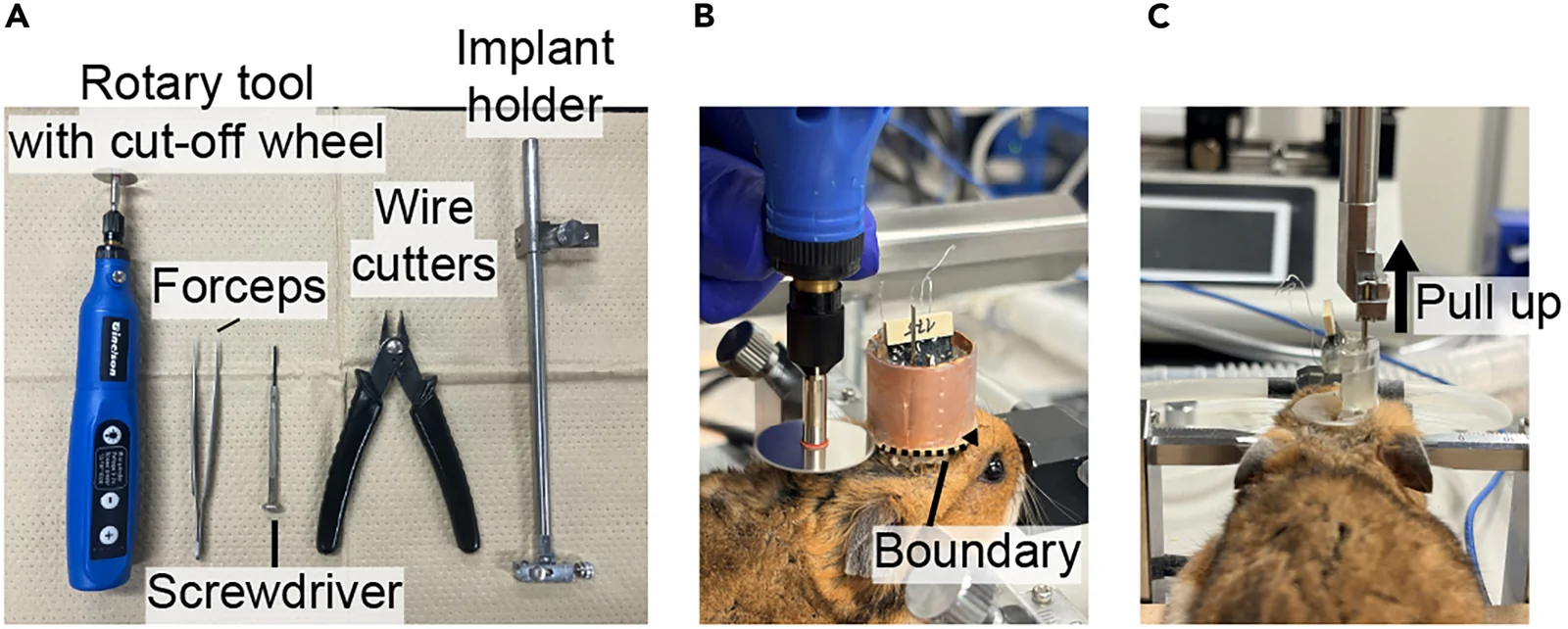
Recovery procedure for reusable silicon probes (A) Tools required for silicon probe recovery. (B) Separation of the boundary between the base and the Faraday cage using a rotary tool with a cut-off wheel. (C) Removal of the drive body and silicon probe from the skull using the implant holder.
104. Fix the head in a stereotaxic apparatus, and ensure the top surface of the microdrive is parallel to the implant holder.

**CRITICAL:** If these are not aligned in parallel, the drive will not be pulled straight up, resulting in probe breakage.

105. Using wire cutters, cut the reference electrode wire and the silicon probe electrode wire that were soldered together within the solder pocket of the Faraday cage (Figure 9B-3).

***Note:*** When reusing the silicon probe, cutting the wires as close as possible to the soldered region improves the likelihood of successful recovery and reuse.

106. Cut along the boundary between the base and the Faraday cage using a cut-off wheel (Figure 12B).

**CRITICAL:** Take care not to insert the rotary wheel deeply into the Faraday cage. This may damage the probe and/or the drive shaft.

***Note:*** If the Faraday cage cannot be detached after cutting around the entire boundary, bedding material or debris may have entered the internal structure. In such cases, the Faraday cage should be carefully broken and removed using wire cutters and forceps.

107. Remove the screws fixed in hole V of the drive body (Figure 9D).
108. Secure the pin attached to hole II of the drive body (Figure 9D) to the implant holder, and slowly lift the drive vertically upward to retrieve the silicon probe (Figure 12C).

***Note:*** Recover the probe before transcardial perfusion to avoid contamination with paraformaldehyde. Reuse the recovered silicon probes after cleaning them overnight with the enzymatic detergent Terg-a-Zyme, followed by rinsing with ultrapure water for 1 h.

***Note:*** We successfully recovered all implanted probes without damage (N = 10/10). In one case, a single shank was accidentally damaged during the cleaning process; however, the remaining shanks on that probe remained completely intact. Importantly, recordings obtained using reused probes exhibited signal quality that was virtually indistinguishable from that of brand-new probes, indicating that the probes maintained their functionality after multiple rounds of use.

## Expected outcomes

Representative EEG/EMG recordings are shown in Figure 13A. EEG signals were recorded and band-pass filtered between 0.5 and 20 Hz. EMG signals were similarly band-pass filtered between 50 and 200 Hz.

**Figure 13:**
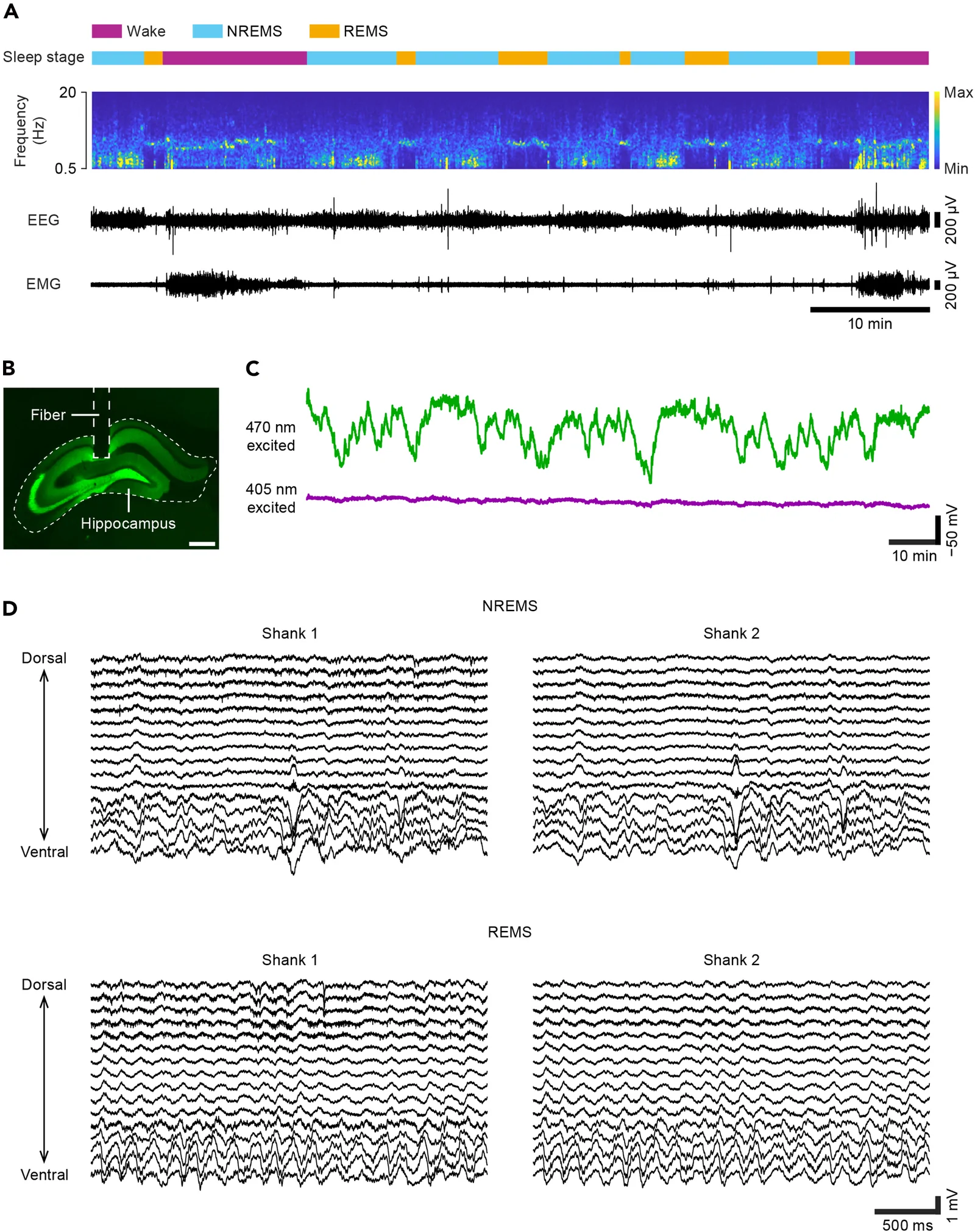
Representative recordings obtained using EEG/EMG, fiber photometry, and silicon probe methods (A) Representative EEG/EMG recording data. The top color bar indicates sleep stages. The middle heatmap shows the power spectrogram of the EEG signal. The bottom traces show EEG and EMG signals. Wake, wakefulness; NREMS, non-rapid eye movement sleep; REMS, rapid eye movement sleep. (B) Representative histological image showing hippocampal expression of the fluorescent sensor GRAB_NE2m following injection of AAV2/9-hSyn-NE2m (4.74 × 10^12^ gc/mL, 300 nL), immunostained with anti-GFP antibody and Alexa Fluor 488. The optical fiber tract and hippocampal region are outlined by dotted lines. Scale bar, 500 μm. (C) Representative fiber photometry recording. Fluorescence signals obtained with 470 nm and 405 nm excitation are shown in green and purple, respectively. (D) Representative silicon probe recordings obtained from two shanks. Left and right panels show recordings from shank 1 and shank 2, respectively. Top and bottom panels show representative local field potentials during NREMS and REMS, respectively.

A representative histological image of a fiber implanted site is shown in Figure 13B. Expression of GRAB_NE2m delivered by AAV2/9-hSyn-NE2m (4.74 × 10^12^ gc/mL, 300 nL) in the hippocampus, as well as the insertion track of the optical fiber cannula, can be confirmed. Because the procedures for transcardial perfusion fixation and immunohistochemistry are generally similar to those commonly used in mice and rats, detailed descriptions of these methods are omitted in this protocol.^5^ A representative fiber photometry recording is shown in Figure 13C. Fluorescence signals obtained using 405 nm and 470 nm excitation were recorded as PMT output voltages (V).

Representative local field potential recordings obtained using a 2-shank silicon probe are shown in Figure 13D. Each shank (shank 1 and shank 2) consisted of 16 recording channels, enabling simultaneous recordings of hippocampal local field potentials. Signals were recorded relative to the reference electrode at 30 kHz, and band-pass filtered between 0.1 and 7,603.8 Hz.

## Quantification and statistical analysis

### Analysis of EEG recording data

EEG and EMG signals were recorded at 3 kHz and downsampled to 1 kHz. A 60 Hz notch filter was applied to remove 60 Hz noise, followed by band-pass filtering (0.5–20 Hz for EEG; 50–200 Hz for EMG). Subsequently, the root mean square (RMS) envelope for both EEG and EMG data was calculated using a 0.5-s sliding window. Periods exceeding 20 times the RMS envelope value were defined as noise, and the corresponding 1.5-s data segments were set to zero. Note that appropriate downsampling rates and filtering parameters vary depending on the analysis purpose, so suitable parameters should be selected accordingly. Frequency analysis was performed using EEG signals, and spectrograms (power spectral density in μV^2^/Hz) were generated using short-time Fourier transform analysis. Sleep stages were classified into wakefulness, rapid eye movement sleep (REMS), and non-REM sleep (NREMS) as follows: periods with higher EMG activity as wakefulness, periods with higher delta band (0.5–4 Hz) power as NREM sleep, periods with a higher theta-delta band power ratio (theta band, 5–12 Hz) as REM sleep, and remaining periods as the preceding stage.

### Analysis of fiber photometry recording data

PMT output voltage signal was recorded at 1 kHz along with 470 and 405 nm excitation illumination timing signals. Fluorescent signals were extracted as the mean signal of individual excitation epochs, resulting in both 20 Hz signals. Subsequently, the extracted signal was smoothed by a 40-point moving average. Motion correction, bleaching correction, and artifact rejection were not performed in this protocol. The necessity and selection of these preprocessing methods should be determined based on the characteristics of the signals and the objectives of the analysis.

### Analysis of LFP recording data

Local field potential (LFP) signals were acquired at 30 kHz with a 0.1–7,603.8 Hz band-pass filter. Behavioral videos were simultaneously recorded at 30 fps, and synchronization between LFPs and video was achieved using TTL pulses generated by the camera shutter.

Sleep and wake states were initially estimated from animal motion quantified by frame subtraction, followed by manual verification using synchronized LFP signals. LFPs were downsampled to 1 kHz and analyzed using short-time Fourier transform to calculate power spectral density and the theta/delta ratio. Epochs were classified as wakefulness, NREMS, or REMS based on movement and spectral characteristics.

The analyses described above were used to generate the representative data shown in this protocol. In addition, the high-quality signals obtained from silicon probes are compatible with standard electrophysiological analysis pipelines widely used in rodents, including frequency-domain analysis, current source density analysis, and spike sorting for single-unit isolation.

## Limitations

The procedures described in this protocol were optimized for adult Syrian hamsters. Application of this protocol to juvenile animals or other hamster species may require adjustments to anesthetic management and stereotaxic coordinates because of differences in body size, skull morphology, and anesthetic sensitivity. In addition, individual variations in skull thickness, muscle development, and dura mater rigidity may affect stereotaxic accuracy and surgical difficulty. The present protocol was primarily validated for recordings from cortical and hippocampal regions. While these approaches are generally robust for relatively superficial brain regions, targeting deeper structures such as the hypothalamus or brainstem may be more challenging because of increased sensitivity to insertion angle, brain displacement, and inter-animal anatomical variability.

## Troubleshooting

[Common].

### Problem 1

The hamster shows signs of pain and begins to awaken during surgery (related to Steps 12–14, 22–30, 39–47, 50–62, and 82–99).

### Potential solution

Administer an additional subcutaneous dose of meloxicam (1 mL/kg) using a 1-mL syringe. Closely monitor the animal after surgery for signs of pain. However, if an additional dose of meloxicam was administered during surgery, avoid administering another dose immediately after surgery. During the days following surgery, provide additional analgesia once daily as needed if signs of pain are observed.

### Problem 2

Bregma and/or lambda are difficult to identify (related to Steps 24, 40, 43, 53, 56, 83, and 88).

### Potential solution

Clean the skull with a saline-moistened cotton swab to remove debris, and then dry it with an air blower to enhance visibility of bregma and lambda.

### Problem 3

Uncontrolled bleeding from the skull surface or muscle (related to Steps 12–14, 22–30, 39–47, 50–62, and 82–99).

### Potential solution

Repeatedly wash the bleeding site with saline using a 1-mL syringe and gently wipe the skull surface or surrounding tissue with a cotton swab to remove blood. If the bleeding does not stop, apply 0.1% adrenaline using a cotton swab and gently press the bleeding site for at least 1 min.

### Problem 4

Transbond or dental acrylic resin adheres to the skin or muscle tissue during surgery (related to Steps 27, 30, 60, and 95).

### Potential solution

If only a small amount of either material adheres to the tissue, it can be peeled off with forceps after curing. If a large amount adheres to the tissue, carefully grind it away using a drill.

### Problem 5

Inflammatory responses, such as pus formation around the adhesive resin cement or dental acrylic resin, occur after chronic implantation (related to Steps 31, 63, and 100).

### Potential solution

Check the implantation site daily after surgery. If pus formation or severe inflammatory responses are observed around the adhesive resin cement or dental acrylic resin, terminate the experiment at an early stage. Histological assessment of tissue responses, including gliosis, should be performed depending on the objectives of the experiment.

[EEG].

### Problem 6

The screw electrode for EEG recording becomes detached from, or loosened in, the skull during surgery (related to Step 27).

### Potential solution

Approximately two full turns are generally sufficient for screw insertion. During the initial insertion, the screw should be rotated while applying gentle downward pressure perpendicular to the skull surface to allow the screw threads to engage the bone. Once the screw is engaged, it can usually be advanced by rotation alone. However, if the screw becomes loose or rotates idly, downward pressure should again be applied while rotating the screw. If the drilled hole is too large, it is recommended to identify another suitable location and repeat the procedure.

### Problem 7

Implantation of the screw electrode causes the wire to twist, reducing the maneuverability of other wires and the pin header (related to Step 27).

### Potential solution

Before implanting the screw electrode, the wire should be slightly twisted in the direction opposite to that used for screw insertion. This compensates for wire twisting after implantation and improves the maneuverability of the wires and pin header.

### Problem 8

The EMG wire cannot be readily inserted into the neck extensor muscles during surgery (related to Step 29).

### Potential solution

The insertion portion of the EMG wire should be kept as straight as possible. Grasping the muscle with forceps and inserting the wire into the grasped muscle facilitates wire insertion.

### Problem 9

The animal moves during the EMG wire insertion (related to Step 29).

### Potential solution

Increase the depth of anesthesia or apply lidocaine locally to help calm the animal.

### Problem 10

The implanted electrode becomes detached after surgery or during recording (related to Steps 30 and 33).

### Potential solution

Detachment may occur when the contact area secured with resin cement is insufficient. To improve stability, remove a larger area of muscle and connective tissue to increase the skull surface available for resin cement application. In addition, older animals may be more susceptible to implant detachment because the skull becomes increasingly laminar and fragile with age. In such cases, selecting younger animals may improve implant stability.

[Fiber photometry].

### Problem 11

Fluorescence signals derived from the fluorescent sensor are weak or undetectable during recording (related to Step 65).

### Potential solution

Insufficient viral expression, inaccurate injection coordinates, reduced viral activity, or incomplete removal of debris from the brain surface during fiber implantation may contribute to this issue. Because expression of the fluorescent sensor may be weak during the initial recording session, fluorescence signals may become detectable after an additional incubation period. If fluorescence signals are still not observed in subsequent recording sessions, inaccurate injection targeting or reduced viral activity should be considered.

### Problem 12

The virus spreads outside the target brain region (related to Step 46).

### Potential solution

Excessive injection volume, overly rapid injection speed, or backflow along the injection track may cause this problem. To prevent viral spread, reduce both the injection volume and injection speed, and keep the glass pipette in place for a certain period after injection. In addition, the glass pipette should be withdrawn slowly.

### Problem 13

The optical fiber cannula becomes loose after surgery (related to Step 60).

### Potential solution

Insufficient cement volume or an inadequate cement fixation area may cause loosening of the optical fiber cannula. Therefore, increasing the amount of cement and expanding the fixation area are recommended. In addition, hamsters may strongly hit their heads against the cage, particularly the ceiling, which can loosen the cannula. Therefore, postoperative housing in a cage with sufficient height is recommended.

[Silicon probe].

### Problem 14

The probe is fixed in a bent position after the epoxy adhesive fully cures (related to Step 76).

### Potential solution

Epoxy adhesives can undergo slight deformation during final curing, which can alter probe alignment. To minimize this issue, temporarily fix the probe using an adhesive with minimal deformation during curing, such as a UV-curable adhesive, before applying the epoxy adhesive.

### Problem 15

The 3D-printed microdrive base (Figure 9B) interferes with the underlying muscle during implantation (related to Step 91).

### Potential solution

Change the orientation of the base during implantation. If the problem persists, redesign the size of the base. If the base bottom size changes, its thickness may also change. During this process, avoid making the shaft of the base excessively thin, as this may interfere with successful 3D printing or increase the risk of breakage when the animal strikes its head against the cage.

### Problem 16

The SPI cable becomes disconnected from the headstage during recording due to the hamster’s strong movements (related to Step 102).

### Potential solution

Cover the connection area with an easily removable silicone-based adhesive (Kwik-Sil) before the recording.

## Resource availability

### Lead contact

Further information and requests for resources and reagents should be directed to and will be fulfilled by the lead contact, Hiroaki Norimoto (norimoto.hiroaki.h3@f.mail.nagoya-u.ac.jp).

### Technical contact

Questions about the technical specifics of performing this protocol should be directed to and will be answered by the technical contacts, Yasutaka Mukai (mukai@nagoya-u.jp) and Hiroaki Norimoto (norimoto.hiroaki.h3@f.mail.nagoya-u.ac.jp).

### Materials availability

This study did not generate new reagents.

### Data and code availability

The STL files for the microdrive design and source data underlying the figures in this paper have been deposited in the Mendeley Data repository: https://doi.org/10.17632/n64gw7ywy4.2.

## Acknowledgments

We thank all the members of the Norimoto Laboratory for their daily discussions and advice, including technical support and suggestions regarding experimental design and research direction. This work was supported in part by Grant-in-Aid for 10.13039/501100000646JSPS Fellows (JP25KJ1411) and JST SPRING (JPMJSP2125) to R.Y.S., 10.13039/501100001691KAKENHI (JP24K18232, JP23KJ0002, JP22K15225, and JP21K20688) and JGC-10.13039/501100004288Saneyoshi Scholarship Foundation to Y.M., KAKENHI (JP23KJ0004 and JP24K18155) to R.K., JST 10.13039/501100009023PRESTO (JPMJPR2048), JST FOREST (JPMJFR224D), JST CREST (JPMJCR24T4), KAKENHI (JP23K18251, JP24K02058, JP24H01996, JP25H02606, JP26H00447), the 10.13039/501100008662Murata Science Foundation, the 10.13039/100008732Uehara Memorial Foundation, the 10.13039/501100005865Mochida Memorial Foundation for Medical and Pharmaceutical Research, a Grant for Basic Science Research Projects from the 10.13039/100008608Sumitomo Foundation, the 10.13039/501100007263Astellas Foundation for Research on Metabolic Disorders, the 10.13039/100032954Nakajima foundation, the 10.13039/100007428Naito foundation, the Toray Science and Technology Grant, Ono Pharmaceutical Foundation for Oncology, Immunology, and Neurology, and 10.13039/100007449Takeda Science Foundation to H. Norimoto.

## Author contributions

Methodology, R.Y.S., Y.M., S.D., and S.I.; investigation, F.Y., R.Y.S., Y.M., H. Nishimura, M.K., and R.K.; project management and conception, H. Norimoto; writing, F.Y., R.Y.S., Y.M., H. Nishimura, and H. Norimoto with input from all authors.

## Declaration of interests

The authors declare no competing interests.
